# Prevalence and Demographic Correlates of “Rough Sex” Behaviors: Findings from a U.S. Nationally Representative Survey of Adults Ages 18–94 Years

**DOI:** 10.1007/s10508-025-03245-9

**Published:** 2025-11-04

**Authors:** Debby Herbenick, Tsung-chieh Fu, Xiwei Chen, Sumayyah Ali, Ivanka Simić Stanojević, Devon J. Hensel, Paul J. Wright, Zoë D. Peterson, Jaroslaw Harezlak, J. Dennis Fortenberry

**Affiliations:** 1 Department of Applied Health Science, Indiana University School of Public Health, Indiana University, SPH 116, Bloomington, IN 47405 USA; 2 Center for Sexual Health Promotion, Indiana University School of Public Health, Indiana University, Bloomington, IN USA; 3 Biostatistics Consulting Center, Indiana University, Bloomington, IN USA; 4 Department of Sociology, Indiana University-Indianapolis, Indianapolis, IN USA; 5 Adolescent Medicine, Indiana University School of Medicine, Indianapolis, IN USA; 6 Communication Science, The Media School, Indiana University-Bloomington, Bloomington, IN USA; 7 Department of Applied Psychology in Education in Research Methodology, School of Education, Indiana University, Bloomington, IN USA; 8 The Kinsey Institute, Indiana University, Bloomington, IN USA; 9 Department of Epidemiology and Biostatistics, Indiana University School of Public Health, Indiana University, Bloomington, IN USA

**Keywords:** Sexual choking, Non-fatal strangulation, Rough sex, Sexual behavior, Population sexual health

## Abstract

Rough sex behaviors have become prevalent among young adults in the U.S. and internationally. However, little is known about these behaviors at the population level. Using nationally representative survey data of 9029 U.S. adults, we aimed to provide population estimates and demographic correlates of 10 such behaviors: consensual and nonconsensual hair pulling, biting, face slapping, genital slapping, light spanking, hard spanking, choking, punching, name-calling, and smothering. We found that 47.8% of women, 60.8% of men, and 67.3% of transgender and gender nonbinary participants (TGNB+) had ever done one or more of the behaviors to a partner. Also, 53.8% of women, 45.7% of men, and 69.5% of transgender and gender nonbinary participants reported that a partner had ever performed at least one type of the assessed behaviors on them, with consent. Experiencing one or more of the behaviors done to them without consent was reported by 19.6% of women, 15.9% of men, and 33.5% of TGNB+ participants. As a general pattern, the behaviors we assessed tended to be more prevalent among younger cohorts as compared with older cohorts and to be reported by more sexual orientation minorities than those identifying as heterosexual. Sexuality educators and clinicians need to be aware of these emerging sexual behaviors. Also, public health agencies should address the increased prevalence of sexual choking, which is usually done as a form of neck compression or strangulation and poses unique risks to health.

## Introduction

Although definitions vary, “rough sex” tends to refer to sexual practices such as hair pulling, biting, spanking, slapping, choking (usually done as a form of neck compression or strangulation), smothering, and name-calling (e.g., usually as a form of degradation), as part of sexual activities (Döring et al., 2024; Gavey & Brewster, 2025; Herbenick et al., 2021a; Tomassilli et al., 2009). Over at least the past decade, some of these sexual practices have become prevalent among young adults in the United States, Australia, Belgium, Germany, Iceland, and Italy (Daminato et al., 2024; Döring et al., 2024; Herbenick et al., 2023a, 2023c; Holvoet et al., 2017; Pavanello Decaro et al., 2024; Sharman et al., 2025a; Vilhjálmsdóttir & Forberg, 2023).

Rough sex behaviors have also become widely represented in social media, television shows, popular songs, erotica, and pornography, which may contribute to perceptions that such practices are erotic, mainstream, pleasurable, and/or expected parts of sex (Balle et al., 2024; Bridges et al., 2016; Herbenick et al., 2022a, 2022b, 2022c; Keene, 2021; Tholander et al., 2022; Wright et al., 2022a, 2023). When rough sex behaviors are portrayed in mainstream culture, they are often presented as “kinky” yet tend to be disconnected from kink or BDSM communities, and by extension, their associated cultural norms and principles related to education, consent, boundaries, and aftercare (Lee et al., 2015; Williams et al., 2014).

In the 2015 Sexual Exploration in America Survey (SEAS)—a U.S. nationally representative survey of adults—42% of women and 40% of men ages 18 to 91 reported finding “rough sex” (a term that was not defined for participants) to be somewhat or very appealing (Herbenick et al., 2017). As is true for many kinds of sex that are both consensual and wanted, including more traditional kinds of sex (see Fortenberry, 2019), people’s decision to participate in sexual practices sometimes described as “rough” or “kinky” may reflect trust and vulnerability between partners and can enhance sexual pleasure (Herbenick et al., 2022b, 2022c, 2023a; Sprott et al., 2021a; Sprott & Williams, 2019). Indeed, in studies about kink more generally, as well as studies of specific sexual practices such as partnered sexual choking, people have described the important roles of trust, intimacy, and vulnerability in their sexual experiences (Herbenick et al., 2022b, 2022c; Hughes & Hammack, 2022; Martin, 2023).

Yet, any kind of sex—whether traditional sex or rough sex—that is done without consent or in situations in which people exceed a partner’s limits or boundaries without permission, can reduce or inhibit sexual pleasure as well as cause or contribute to psychological and/or physical harm (Bowling et al., 2022; Herbenick et al., 2019; Wright et al., 2022b). Even wanted and consensual kink and rough sex experiences may result in physical injuries, and in rare cases, death (Herbenick et al., 2022d; Pinson, 2019; Schori et al., 2022; Sprott et al., 2021b). As rough sex has become more prevalent and part of mainstream culture, it has also sometimes made people (and especially young women) more vulnerable to physical and sexual violence, with resulting claims of “rough sex gone wrong” (Bows & Herring, 2020) or with injuries sometimes being unclear as to whether they were caused by consensual sexual choking/strangulation or from strangulation occurring from partner violence (Marr & Bugeja, 2025). Given the potential for rough sex behaviors to contribute positively to people’s sexual pleasure and intimacy, as well as the potential to cause significant harm, it is important for public health and sexual health professionals to understand more about these so-called “rough sex” practices, including who is engaging in them.

### Prevalence of Rough Sex

Due to public health priorities related to pregnancy and sexually transmitted infections, U.S. surveillance surveys that have addressed sexual practices—such as the National Survey of Family Growth, the Youth Risk Behavior Survey, and the National Health and Nutrition Examination Survey—have tended to limit their assessment to sexual behaviors such as oral sex, vaginal intercourse, and anal intercourse (Centers for Disease Control & Prevention, 2024a, 2024b, n.d.; National Center for Health Statistics, n.d.-a, n.d.-b). Around the world, including in the U.S., United Kingdom, Australia, Germany, and Norway, population-representative surveys that have focused on sexual health have assessed somewhat more diverse forms of sexual expression, including masturbation, chest/breast stimulation, manual-genital stimulation, and/or the use of sexual enhancement products (Cerwenka et al., 2021; Fischer et al., 2022; Gerressu et al., 2008; Herbenick et al., 2010; Richters et al., 2003; Traeen et al., 2023). Yet, few nationally representative surveys have assessed practices that might be considered forms of rough sex or kinky sex.

As one example, the first Australian Study of Health and Relationships (ASHR-1), conducted in 2001 and 2002, used computer-assisted telephone interviews to conduct a nationally representative study of Australians ages 16 to 59. The researchers assessed experience with BDSM practices and found that 2% of men and 1% of women reported having engaged in BDSM within the prior year (Richters et al., 2003). However, the study was limited to the assessment of past year experience (not lifetime experience) and only asked about BDSM experiences broadly rather than specific BDSM practices; thus, it is not clear to what extent these BDSM behaviors might correspond to contemporary ideas of rough sex. In the same study, the researchers also asked participants whether they had engaged in fisting in the prior year, though did not define this behavior; fewer than 1% of participants had done so (Richters et al., 2003).

As a second example, the 2015 SEAS reported on the prevalence, by gender and age, of more than thirty sexual practices among a nationally representative survey of 2021 U.S. adults ages 18–91 (Herbenick et al., 2017). Relevant to the kinds of sex commonly associated with kink or rough sex, the researchers found that 15% of participants had playfully whipped a partner or been playfully whipped and 32% had spanked or been spanked as part of sex. The study was limited to assessing sexual practices more generally (e.g., spanking/being spanked), rather than in separate giving (spanking) and receiving (being spanked) roles.

As a third example, the 2016 National Survey of Pornography, Relationships, and Sexual Socialization (NSPRSS) collected data from a U.S. nationally representative sample of adults ages 18 to 60 and reported on the prevalence of spanking, sexual choking, name-calling (e.g., calling someone a bitch, slut, or whore), and other behaviors that were conceptualized as part of “pornographic sexual scripts” (Herbenick et al., 2020). The researchers found that more men reported “doing” (performing) each of these sexual practices and more women reported being on the receiving end of each. For example, 77% of men and 54% of women had ever spanked a partner whereas 46% of men and 66% of women had ever been spanked. Similarly, although 20% of men and 12% of women reported having choked a partner during sexual activities, nearly the opposite was true for having ever been choked which was reported by 11% of men and 21% of women. And although 23% of men and 12% of women reported calling a partner names during sex (such as slut, whore, or bitch), 14% of men and 26% of women reported that a partner had called them such names during sex (Herbenick et al., 2020). Yet, the study was limited to 18- to 60-year-olds and assessed a narrow range of behaviors.

As a fourth example, the 2021 National Survey of Sexual Wellbeing surveyed a U.S. nationally representative sample of 2525 adults ages 18–88. The researchers found that 35% of women ages 18–24 and 11% of women ages 25–29 reported that their partner had choked them during their most recent consensual sexual encounter (Herbenick et al., 2023a). In comparison, just 3% of men ages 18–29 reported having been choked at their most recent consensual sexual encounter. Younger adults were more likely to report engaging in choking. Also, more women than men reported that their partner had choked them. However, no other event-level rough sex behaviors were assessed.

Several national online panel surveys have also addressed kinky or rough sex practices. In a 2017 study in Belgium, 1027 adults ages 18–65 completed an online survey, with 20–30% reporting interest or engagement in hitting or being hit during sex, about 10% reporting interest or engagement in verbal humiliation, and less than 10% indicated that they had ever engaged in or fantasized about controlling the breathing of one’s partner (Holvoet et al., 2017). In a 2022 survey conducted in Germany, researchers surveyed 1101 adults ages 18 to 69 who were members of an opt-in panel and found that 29% reported having ever engaged in some kind of “rough sex” (the term was not defined for participants) and that adults younger than 40 were significantly more likely to report engaging in rough sex as compared to adults ages 40 and older (Döring et al., 2024). In an online panel survey of 4702 Australians ages 18–35 who were “sexually experienced” (also not defined for participants), the researchers found that about half of participants had ever choked/strangled a partner or been choked/strangled during sexual activities (Sharman et al., 2025a). They also found that women and transgender/gender nonbinary (TGNB) participants were more likely to report being choked/strangled during sex and those men and TGNB participants were more likely to report that they had choked/strangle their partner(s) during sexual activities. Taken together, these studies suggest that rough sex behaviors have been observed in sizable proportions around the world; however, these findings are tempered by the data being from non-probability samples, which are limited in their generalizability and some of which may be challenged by data quality issues (Douglas et al., 2023; Kan & Drummey, 2018; Lehdonvirta et al., 2021; Peer et al., 2021).

Additionally, a series of campus-representative surveys at a single public U.S. public university has found that more than 80% of college students with a sex partner reported having engaged in rough sex (Herbenick et al., 2021a). Further, they found that half of students having choked and/or been choked during sex, with women and TGNB students more likely than men to report being choked, spanked, and called names during sexual activities (Herbenick et al., 2021b, 2022d, 2023c).

### Demographic Correlates of Rough Sex

The limited data on rough sex from nationally representative surveys, taken together with data from national online convenience surveys, non-probability samples, and college campus-representative surveys, suggests several demographic correlates with rough sex. In terms of age, younger adults (generally those younger than 40 or 50) tend to be more likely than older adults to report having ever engaged in various forms of rough sex, which has been found in both nationally representative (Herbenick et al., 2020, 2023a) and various other surveys (Daminato et al., 2024; Döring et al., 2024; Sharman et al., 2025a; Vilhjálmsdóttir & Forberg, 2023). In terms of gender, women tend to be more likely to report being on the receiving end of rough sex practices and men tend to be more likely to report enacting rough sex on their partner(s), though transgender and gender nonbinary participants tend to report high rates of both giving and receiving rough sex (Döring et al., 2024; Herbenick et al., 2021a, 2023a; Sharman et al., 2025a, 2025b). In terms of sexual orientation identity, there is a paucity of data from population-level surveys. However, college representative surveys (Herbenick et al., 2021a, 2021b), convenience surveys (Sharman et al., 2025b), and online non-probability surveys (Sharman et al., 2025a), suggest greater engagement in rough sex practices by sexual orientation minorities as compared to heterosexual individuals.

However, the extant literature has several notable gaps. First, there is still relatively little diversity in the sexual practices assessed, with recent studies tending to focus on sexual choking due to its rapid increase in prevalence and its higher level of risk compared to other rough sex behaviors (Herbenick et al., 2022d; Sharman et al., 2025b; Vilhjálmsdóttir & Forberg, 2023). Second, the little population-level data available on diverse sexual practices are now somewhat old, as the ASHR-1 was conducted in the early 2000s, the SEAS was conducted in 2015, and the NSPRSS was conducted in 2016 (Herbenick et al., 2017, 2020; Richters et al., 2003). Considering that at least some rough sex practices appear to be increasing in prevalence and have also increased as part of sexual assault cases (Cannon et al., 2020; Valentine et al., 2023), and even implicated in homicides (Bows & Herring, 2020), ongoing surveillance studies are needed to track rough sex behaviors at the population level. Third, the sexual practices that have been assessed have not always been asked about in terms of the role that people adopt (e.g., spanking vs. being spanked or choking vs. being choked; Herbenick et al., 2017). Fourth, although nonconsensual rough sex has been mentioned in qualitative interviews, college surveys, convenience surveys, and open-ended items from one U.S. nationally representative survey (Herbenick et al., 2019, 2022b, 2022c; Keene, 2021; Sharman et al., 2025b), most studies to date have not empirically assessed rough sex in the context of consent. That is, there are currently no nationally representative survey data that speak to the prevalence of having experienced specific rough sex practices without permission or consent.

Finally, few nationally representative studies have assessed people’s engagement in rough sex practices, and given the often relatively small sample sizes in studies of rough sex, data have not often been presented separately for sexual minoritized individuals (Herbenick et al., 2017; Holvoet et al., 2017). Even less is known, via nationally representative probability surveys, about engagement in rough sex practices by gender minoritized people. Often even when studies do include gender minoritized participants, the sample size is too small to draw meaningful conclusions. Yet, vulnerability to nonconsensual rough sex might differ for certain gender minorities. As one example, perhaps those assigned female at birth are particularly vulnerable to nonconsensual sex due to their femininized socialization, or alternatively, perhaps those that are assigned male but present as more feminine are more vulnerable because they may be punished for violating traditional masculinity norms. The present study addresses each of these gaps.

As rough sex practices appear to have become more prevalent and have both positive and negative associations with human health and well-being, it is critical that public health researchers include these practices in assessments of sexual behavior. As surveillance is a core function of public health, the present study is surveillance focused and atheoretical in nature. Using U.S. nationally representative survey data of adults ages 18 and over, we aimed to provide population estimates of engaging in hair pulling, biting, face slapping, genital slapping, light spanking, hard spanking, choking, punching, name-calling, and smothering and to examine these in relation to gender, age group, sexual orientation identity, role (having done these to a partner and having had someone do these behaviors to them), and consent.

## Method

### Participants

Data are from the 2022 National Survey of Sexual Health and Behavior (NSSHB), which is the 8th wave of the NSSHB (“NSSHB-8”), a series of U.S. nationally representative surveys that have been conducted since 2009. During December 2022 and January 2023, NSSHB-8 data were collected through confidential online surveys sent to members of the Ipsos KnowledgePanel®, which is commonly used for U.S. nationally representative data collection on a wide range of topics, including health and sexual health topics (Kyanko et al., 2020; Mark et al., 2024; Scherer et al., 2021; Semenza et al., 2024; Walsh-Buhi et al., 2024). KnowledgePanel® was established through address-based sampling via the U.S. Postal Service’s Delivery Sequence File (i.e., the panel is established via probability methods and is not an “opt-in” panel). Once Ipsos identifies individuals’ addresses to be sampled, they send invitation letters, reminder postcards, and use follow-up telephone calls to recruit individuals and their household members into the panel. Households that do not have internet access may be provided with a web-enabled device and free internet to facilitate KnowledgePanel® participation. The panel is comprised of about 60,000 non-institutionalized U.S. adults. KnowledgePanel® members complete surveys for which they receive points that may accumulate over time and be exchanged for cash or merchandise.

To minimize participant burden, KnowledgePanel® members are recruited for only up to a few surveys in any given month. For NSSHB-8, Ipsos first identified a sampling frame of 14,019 adult KnowledgePanel® members ages 18 and over. Ipsos then sent these individuals a message, either via email or through their online dashboard that let them know a new survey was available. Those who clicked on the link were provided with detailed information about the study, which was described as being about people’s “sexual health and experiences” and being for all people “regardless of whether they have ever even kissed anyone or had sex.” Individuals could electronically indicate their consent to participate and then proceed to complete the confidential online survey. The survey was available in English and Spanish languages and took a median of 21 min to complete.

Of the 14,019 adults in the sampling frame, 8742 (62.4%) consented to participate in the survey and 8666 provided complete surveys, for a 61.8% completion rate. Once the data were collected, Ipsos then created statistical weights to account for any differential nonresponse (i.e., under-coverage or over-coverage of certain demographic groups). For the NSSHB-8, statistical weights were based on population-specific demographic distributions based on the 2021 Current Population Survey and accounted for age by gender, Census region, Metropolitan status, household income, race, ethnicity, income, ethnicity, education, and language proficiency by age. Ipsos then sent a de-identified dataset to our research team; at no point did we interact directly with participants or have access to their names or contact information.

The teen and adult participants were weighted to reflect their correct proportions within total participants, which was about 6.5% for teens and 93.5% for adults. There were a total of 9683 participants, including both adults and adolescents. Due to the large number of rough sex behaviors, we assessed as well as the sociosexual and developmental differences between adults and adolescents, the present analysis focuses only on the adults, for which there was a weighted base of 9053 adult participants. Results from the adolescent sample will be presented in a subsequent paper. Of the 9053 adult participants, those without gender identity data (*n* = 37) or who had incomplete data on the analytic items were not included in the present analyses, leaving a weighted sample of 9029 individuals.

Participants ranged in age from 18 to 94 years (*M*_age_ = 48.19, SD = 17.68, median = 48.0). About half of participants described themselves as women (50.5%, *n* = 4562), with 48.4% describing themselves as men (*n* = 4346), and 1.3% (*n* = 121) identifying as transgender, gender nonbinary, or in other gender non-conforming ways (TGNB+). For those who described themselves as women, 0.2% (*n* = 10) reported that they had been assigned male at birth. Among those who described themselves as men, 0.4% (*n* = 17) had been assigned female at birth.

In terms of race and ethnicity, 62.1% identified as White (*n* = 5607), 17.1% as Hispanic (*n* = 1542), and 12.0% as Black (*n* = 1079). Most (89.5%, *n* = 8011) described themselves as heterosexual or straight, 4.6% (*n* = 412) as bisexual or pansexual, and 3.3% (*n* = 297) as gay or lesbian. More than half of participants were married (55.4%, *n* = 5000). Also, 55.5% (*n* = 5014) reported a household income of $75,000 or greater. See Table 1 for additional demographic characteristics for the total sample and by gender.

**Table 1:** Participant characteristics of the weighted sample (total and by gender)

	Total				
	*N* = 9029				
	Overall	Women	Men	TGNB+	*p*-value
*Gender*	*% (n)*	*% (n)*	*% (n)*	*% (n)*	
Women	50.5 (4562)	100.0 (4562)	0.0 (0)	0.0	
Men	48.1 (4346)	0.0 (0)	100.0 (4346)	0.0	
TGNB+	1.3 (121)	0.0 (0)	0.0 (0)	100.0 (121)	
*Age*					< 0.001
18–24	9.2 (833)	8.1 (372)	9.8 (425)	30.5 (37)	
25–29	10.7 (965)	10.8 (494)	10.2 (443)	23.0 (28)	
30–39	15.8 (1426)	15.6 (711)	15.9 (689)	21.3 (26)	
40–49	17.2 (1556)	16.7 (760)	18.1 (785)	8.7 (11)	
50–59	16.9 (1524)	16.8 (768)	17.1 (743)	11.0 (13)	
60–69	16.5 (1491)	17.6 (804)	15.7 (681)	4.9 (6)	
70 +	13.7 (1234)	14.3 (653)	13.3 (580)	0.5 (1)	
*Race or ethnic group*					0.169
White	62.1 (5607)	61.7 (2815)	62.7 (2725)	55.2 (67)	
Black	12.1 (1079)	12.7 (579)	11.3 (491)	7.8 (10)	
Hispanic	17.1 (1542)	16.6 (758)	17.4 (756)	22.9 (28)	
Other	8.9 (801)	9.0 (409)	8.6 (374)	14.0 (17)	
*Sexual orientation*					< 0.001
Heterosexual	89.5 (8011)	89.8 (4057)	91.3 (3933)	16.8 (20)	
Gay or Lesbian	3.3 (297)	2.0 (90)	4.3 (187)	17.3 (21)	
Bisexual/Pansexual	4.6 (412)	6.0 (271)	2.3 (100)	34.2 (41)	
Something else	2.5 (225)	2.2 (101)	2.0 (86)	31.8 (39)	
*Geographic region*					0.587
Northeast	17.4 (1569)	17.7 (807)	17.2 (748)	11.5 (14)	
Midwest	20.7 (1865)	20.6 (938)	20.8 (904)	18.9 (23)	
South	38.2 (3451)	38.4 (1753)	38.0 (1651)	38.8 (47)	
West	23.8 (2145)	23.3 (1064)	24.0 (1043)	30.8 (37)	
*Education completed*					0.001
Less than high school	9.4 (847)	8.9 (405)	10.0 (434)	6.1 (7)	
High school	29.1 (2624)	27.5 (1255)	30.9 (1343)	22.1 (27)	
Some college	26.6 (2405)	27.2 (1239)	25.7 (1117)	40.7 (49)	
College degree or higher	34.9 (3153)	36.5 (1664)	33.4 (1452)	31.1 (38)	
*Marital status*					< 0.001
Married	55.4 (5000)	54.5 (2486)	57.1 (2483)	26.1 (32)	
Widowed	4.1 (372)	5.9 (268)	2.3 (100)	3.1 (4)	
Divorced	9.2 (833)	10.8 (492)	7.6 (330)	8.6 (10)	
Separated	1.6 (148)	1.7 (78)	1.5 (65)	4.4 (5)	
Never married	29.6 (2677)	27.1 (1238)	31.5 (1369)	57.8 (70)	
*Annual income ($)*					< 0.001
< 25,000	12.5 (1127)	13.9 (634)	10.9 (473)	16.9 (21)	
25,000—49,999	16.2 (1459)	16.8 (768)	15.3 (664)	21.8 (27)	
50,000—74,999	15.8 (1429)	15.8 (721)	15.8 (685)	18.5 (22)	
> = 75,000	55.5 (5014)	53.5 (2439)	58.1 (2524)	42.8 (52)	

TGNB+ refers to participants who identified as transgender, gender nonbinary, or in other gender non-conforming ways

### Measures

The study questionnaire was developed by an interdisciplinary team with backgrounds in public health, epidemiology, sociology, health communication, clinical psychology, adolescent medicine, biostatistics, sexuality research, and sexuality education. The NSSHB-8 questionnaire includes measures from prior waves of the NSSHB as well as measures from other studies that were modified for use in the NSSHB-8. We then conducted cognitive testing (e.g., interviewing people and using think-aloud procedures) on the questionnaire to improve the clarity and comprehension of the items. The present study focuses on questionnaire items related to background characteristics and engagement in certain rough sex practices, though the term “rough sex” was never used directly with participants. Rather, as noted below, we asked about each behavior.

#### Background Characteristics

Ipsos asks individuals to respond to a number of demographic items when they join KnowledgePanel®; many demographic items are routinely asked again and updated as part of ongoing retention efforts as part of their profile data. These background items were then included as part of the de-identified dataset; these include age, race, ethnicity, education, marital status, geographic area, and household income. Additionally, informed by principles put forth by Beischel et al. (2023), we asked participants about their sex assigned at birth (male, female, not listed with an option to describe), the gender category for which their data should be analyzed (women, men, gender nonbinary, transgender women, transgender men, use a different term with the option to describe), and their sexual orientation identity (asexual, bisexual, lesbian or gay, pansexual, queer, straight/heterosexual, or something else with the option to describe).

#### Performing/Enacting Rough Sex

Using items modified from the core sexual behavior items from previous NSSHB waves (Herbenick et al., 2010), other U.S. nationally representative surveys (Herbenick et al., 2020), and from college campus representative surveys about rough sex practices, Herbenick et al., 2021b), we asked “How recently have you done the following to a partner while you were kissing or having sex together?” and presented a list of these sexual practices: pulled their hair; bit them; slapped their face; slapped their genital area (clitoris, vaginal area, penis, or scrotum); spanked them lightly; spanked them hard enough to leave a mark; choked them (e.g., you used your hands, arm, or object to press against or squeeze a partner’s neck); punched them (hit with a closed fist, whether light or hard) called your partner names like bitch, slut, whore, fag, etc., and smothered them during sexual activities (e.g., you put a pillow or your hand over their mouth/nose). Response options were: In the past month, in the past year, more than a year ago, never done this.

#### Experienced Rough Sex with Permission or Consent

We asked, “How recently has a partner done the following to you with permission or consent while you were kissing or having sex together?” They were then shown the text, “With my permission or consent, someone has…” and a list of the sexual practices and response options described above.

#### Experienced Rough Sex without Permission or Consent

We asked, “How recently has a partner done the following to you without your permission or consent while you were kissing or having sex together?” They were then shown the text, “Without my permission or consent, someone has…” as well as the sexual practices and response options described above.

### Statistical Analysis

We used R version 4.3.1 for statistical analyses described herein and the weighted data were used for all analyses described here. We used descriptive analyses to examine the distributions of baseline and behavioral characteristics across gender groups (women, men, TGNB+). We estimated the past year and lifetime prevalence of rough sex behaviors among adults (age 18 +) by gender (women, men, TGNB+), age group (18–24, 25–29, 30–39, 40–49, 50–59, 60–69, and 70 +), sexual orientation identity (heterosexual, lesbian/gay, and bisexual/pansexual), and—for TGNB+ participants—a comparison of those who had been assigned female at birth (AFAB) versus assigned male at birth (AMAB); for each of these, we used weighted Chi-squared tests for group comparisons. As detailed above, we examined participants’ reports of ten sexual behaviors: hair pulling, biting, face slapping, genital slapping, light spanking, hard spanking, choking, punching, name-calling, and smothering. These are presented separately by gender for behaviors that the participant had done to their partner(s), behaviors a person had done to them with permission or consent, and behaviors a person had done to them without permission or consent. Additional variables were created that reflect whether a person had experienced any of the behaviors within each category (i.e., enacted any of the listed behaviors on a partner; experienced any of these with consent; experienced any of these without consent). Reported *p*-values were two-tailed and nominal significance level was set at < 0.05.

## Results

### Women’s Reports of Enacting Rough Sex Behaviors

As shown in Table 2, 47.8% of women (95% CI: 46.1%, 49.5%) reported having ever done one or more of the listed sexual behaviors to a partner: pulled their hair, bit them, slapped their face, slapped their genital area, spanked lightly, spanked hard enough to leave a mark, choked them, punched them, called them names, or smothered them during sexual activities. Also, 29.5% of women (95% CI: 27.9%, 31.1%) reported having done one or more of these to a partner in the prior year. The five most commonly reported lifetime behaviors were: biting (31.2%), light spanking (29.5%), hair pulling (27.4%), choking (9.2%), and genital slapping (8.6%).

**Table 2:** Women's past year and lifetime rough sex experiences by sexual orientation identity and age

	Overall	Sexual Orientation Identity			*p*-value	Age							*p*-value
		Heterosexual	Bisexual/Pansexual	Lesbian or Gay		18–24	25–29	30–39	40–49	50–59	60–69	70 +	
	*N* = 4562	*N* = 4057	*N* = 271	N = 90		N = 372	*N* = 494	*N* = 711	*N* = 760	*N* = 768	*N* = 804	*N* = 653	
*Participant did this to a partner*													
*Pulled their hair*													
Past year	14.9% (13.7%, 16.3%)	13.1% (11.8%, 14.4%)	39.2% (33.1%, 45.7%)	23.3% (15.4%, 33.6%)	< 0.001	26.2% (20.1%, 33.3%)	31.6% (26.2%, 37.6%)	28.7% (25%, 32.6%)	18.2% (15.3%, 21.6%)	7.5% (5.6%, 9.9%)	1.9% (1.1%, 3.2%)	1.4% (0.7%, 2.9%)	< 0.001
Lifetime	27.4% (25.9%, 29%)	24.9% (23.3%, 26.5%)	57.9% (51.4%, 64.1%)	43.1% (33.3%, 53.6%)	< 0.001	31% (24.5%, 38.3%)	44.8% (38.8%, 51%)	44.9% (40.7%, 49.1%)	37.1% (33.2%, 41.1%)	23% (19.9%, 26.4%)	11.1% (9%, 13.5%)	6.3% (4.6%, 8.5%)	< 0.001
*Bit them*													
Past year	17.1% (15.8%, 18.5%)	15.1% (13.7%, 16.5%)	45.8% (39.5%, 52.3%)	21.3% (14.2%, 30.7%)	< 0.001	27.3% (21.1%, 34.4%)	35% (29.5%, 41.1%)	34.6% (30.7%, 38.8%)	20.6% (17.4%, 24.1%)	7.8% (5.9%, 10.3%)	3.1% (2%, 4.8%)	2.2% (1.3%, 3.8%)	< 0.001
Lifetime	31.2% (29.6%, 32.8%)	28.7% (27%, 30.4%)	63.6% (57.1%, 69.7%)	47.7% (37.5%, 58%)	< 0.001	31.6% (25.1%, 38.9%)	48.9% (42.8%, 55%)	52.3% (48%, 56.5%)	41.9% (37.9%, 46.1%)	25.4% (22.2%, 29%)	13.6% (11.3%, 16.3%)	9.2% (7.2%, 11.6%)	< 0.001
*Slapped their face*													
Past year	2.5% (2%, 3.1%)	2.1% (1.6%, 2.7%)	6.8% (4.2%, 11%)	2.9% (1%, 8.6%)	< 0.001	3.1% (1.4%, 6.7%)	5.7% (3.3%, 9.6%)	6.1% (4.4%, 8.5%)	2.2% (1.2%, 3.8%)	0.4% (0.2%, 1.2%)	0.2% (0.1%, 1%)	1.1% (0.5%, 2.3%)	< 0.001
Lifetime	5.2% (4.5%, 6%)	4.4% (3.7%, 5.3%)	14.5% (10.6%, 19.5%)	7.2% (3.5%, 14.3%)	< 0.001	6.0% (3.3%, 10.6%)	11.3% (7.8%, 15.9%)	10.9% (8.6%, 13.8%)	6.0% (4.4%, 8.2%)	2.1% (1.2%, 3.5%)	1.2% (0.6%, 2.3%)	1.2% (0.6%, 2.5%)	< 0.001
*Slapped their genital area*													
Past year	5.1% (4.3%, 5.9%)	4.7% (3.9%, 5.5%)	9.1% (5.9%, 13.6%)	7.3% (3.8%, 13.6%)	0.004	3.3% (1.5%, 7%)	9.4% (6.3%, 13.8%)	9.9% (7.7%, 12.8%)	7.7% (5.8%, 10.3%)	2.9% (1.9%, 4.5%)	1.4% (0.7%, 2.7%)	1.1% (0.5%, 2.3%)	< 0.001
Lifetime	8.6% (7.7%, 9.6%)	7.9% (6.9%, 8.9%)	15.0% (11%, 20.1%)	17.3% (11.1%, 26%)	< 0.001	4.1% (2%, 8.2%)	12.9% (9.3%, 17.7%)	15.3% (12.5%, 18.7%)	13% (10.4%, 16.1%)	7% (5.3%, 9.1%)	4.5% (3.2%, 6.2%)	2.2% (1.3%, 3.7%)	< 0.001
*Spanked them lightly*													
Past year	18% (16.6%, 19.4%)	16.7% (15.3%, 18.2%)	32.9% (27.2%, 39.2%)	26.2% (17.8%, 36.8%)	< 0.001	14.1% (9.7%, 20%)	33.2% (27.7%, 39.3%)	33.2% (29.3%, 37.4%)	25.1% (21.6%, 28.9%)	12.5% (10%, 15.6%)	6.1% (4.5%, 8.2%)	3.5% (2.3%, 5.5%)	< 0.001
Lifetime	29.5% (27.9%, 31.1%)	27.7% (26.1%, 29.4%)	47.7% (41.4%, 54.1%)	46.3% (36.2%, 56.8%)	< 0.001	15.6% (11%, 21.6%)	38.9% (33.1%, 45.1%)	44.5% (40.3%, 48.7%)	42.2% (38.1%, 46.4%)	33.6% (29.9%, 37.6%)	16.4% (13.9%, 19.3%)	8.9% (6.9%, 11.4%)	< 0.001
*Spanked hard enough to leave a mark*													
Past year	4.3% (3.7%, 5.1%)	3.3% (2.7%, 4.1%)	13.9% (9.9%, 19.2%)	15.8% (9%, 26.2%)	< 0.001	4.6% (2.4%, 8.8%)	11.1% (7.8%, 15.5%)	8.7% (6.7%, 11.3%)	4.8% (3.4%, 6.9%)	1.6% (0.9%, 2.9%)	0.9% (0.4%, 2.1%)	0.9% (0.3%, 2.3%)	< 0.001
Lifetime	8.5% (7.6%, 9.5%)	7% (6.1%, 8%)	22.9% (18%, 28.7%)	27.4% (18.7%, 38.3%)	< 0.001	6.5% (3.7%, 11%)	16% (12.1%, 20.9%)	15.3% (12.5%, 18.4%)	11% (8.7%, 13.9%)	6.5% (4.9%, 8.6%)	3.4% (2.3%, 5.1%)	2% (1.1%, 3.5%)	< 0.001
*Choked them*													
Past year	6.2% (5.4%, 7.2%)	4.8% (4%, 5.8%)	22.1% (17.1%, 28.1%)	17.6% (10.6%, 27.7%)	< 0.001	17.3% (12.4%, 23.6%)	17.3% (13.1%, 22.3%)	11.5% (9.1%, 14.5%)	4.9% (3.4%, 7%)	0.9% (0.4%, 1.9%)	0.5% (0.2%, 1.2%)	0.6% (0.2%, 1.6%)	< 0.001
Lifetime	9.2% (8.3%, 10.3%)	7.5% (6.5%, 8.6%)	31.0% (25.4%, 37.2%)	20.9% (13.4%, 31.2%)	< 0.001	19.1% (14%, 25.4%)	24.0% (19.2%, 29.5%)	17.5% (14.6%, 20.8%)	9.8% (7.6%, 12.5%)	2.9% (1.9%, 4.4%)	0.6% (0.2%, 1.3%)	0.6% (0.2%, 1.6%)	< 0.001
*Punched them*													
Past year	1.0% (0.7%, 1.4%)	0.9% (0.6%, 1.4%)	1.9% (0.8%, 4.6%)	2.9% (0.9%, 9%)	0.067	0.7% (0.2%, 3.1%)	1.3% (0.5%, 3.5%)	2.4% (1.4%, 4.2%)	1.5% (0.8%, 3%)	0.1% (0%, 1%)	0.1% (0%, 0.8%)	0.8% (0.3%, 2%)	0.001
Lifetime	1.8% (1.4%, 2.3%)	1.7% (1.3%, 2.3%)	3.2% (1.6%, 6.3%)	4% (1.5%, 10.5%)	0.065	1.1% (0.3%, 3.6%)	3.3% (1.7%, 6.4%)	3.2% (2%, 5%)	2.7% (1.6%, 4.5%)	0.7% (0.2%, 1.9%)	0.7% (0.3%, 1.6%)	1% (0.4%, 2.2%)	0.001
*Called them names*													
Past year	3.3% (2.7%, 4.1%)	2.9% (2.3%, 3.7%)	7.2% (4.4%, 11.5%)	9.8% (5.2%, 17.8%)	< 0.001	5.6% (3%, 10.4%)	6.9% (4.3%, 10.9%)	6.3% (4.5%, 8.8%)	4.1% (2.7%, 6.3%)	0.9% (0.4%, 1.9%)	1.2% (0.6%, 2.5%)	0.5% (0.1%, 1.5%)	< 0.001
Lifetime	5.9% (5.1%, 6.8%)	5.3% (4.5%, 6.2%)	11.8% (8.3%, 16.6%)	17.1% (10.7%, 26.1%)	< 0.001	7% (4.1%, 11.9%)	9.2% (6.2%, 13.6%)	9.4% (7.2%, 12.1%)	7.8% (5.8%, 10.4%)	4.2% (2.9%, 5.9%)	3.1% (2.1%, 4.6%)	2.1% (1.2%, 3.5%)	< 0.001
*Smothered them during sexual activities*													
Past year	1.4% (1%, 1.9%)	1% (0.7%, 1.5%)	4.1% (2.2%, 7.8%)	5.7% (2.3%, 13.2%)	< 0.001	2.7% (1.2%, 6.3%)	2.7% (1.3%, 5.5%)	2.6% (1.5%, 4.6%)	1.7% (0.9%, 3.3%)	0.2% (0.1%, 1%)	0.2% (0.1%, 1%)	0.6% (0.2%, 1.6%)	< 0.001
Lifetime	2.4% (1.9%, 3.1%)	1.8% (1.4%, 2.4%)	7% (4.3%, 11.1%)	10.1% (4.9%, 19.8%)	< 0.001	4.6% (2.4%, 8.6%)	4.8% (2.8%, 8.2%)	4.3% (2.9%, 6.5%)	3.2% (2%, 5.1%)	0.7% (0.3%, 1.5%)	0.6% (0.3%, 1.6%)	0.7% (0.3%, 1.7%)	< 0.001
*Did any of these to a partner*													
Past year	29.5% (27.9%, 31.1%)	27.4% (25.8%, 29.1%)	59.4% (53%, 65.5%)	30.7% (21.9%, 41.1%)	< 0.001	36.5% (29.7%, 44%)	52.5% (46.3%, 58.6%)	50.9% (46.7%, 55.1%)	40.9% (36.9%, 45.1%)	20.1% (17%, 23.6%)	9% (7.1%, 11.4%)	6.4% (4.7%, 8.6%)	< 0.001
Lifetime	47.8% (46.1%, 49.5%)	45.6% (43.8%, 47.4%)	76.6% (70.4%, 81.9%)	62% (51.8%, 71.3%)	< 0.001	40.5% (33.5%, 48%)	65.8% (59.6%, 71.5%)	67.3% (63.2%, 71.2%)	66% (61.9%, 69.8%)	47.5% (43.5%, 51.5%)	27.6% (24.5%, 31%)	19.5% (16.7%, 22.8%)	< 0.001
*Done to participant with consent*													
*Hair was pulled*													
Past year	20.8% (19.4%, 22.3%)	18.7% (17.2%, 20.2%)	54% (47.7%, 60.3%)	22.4% (14.8%, 32.5%)	< 0.001	31.3% (24.7%, 38.6%)	44.6% (38.6%, 50.8%)	38.9% (34.9%, 43.1%)	27.4% (23.8%, 31.2%)	11.2% (9%, 13.9%)	3.2% (2.1%, 4.9%)	1.4% (0.7%, 2.7%)	< 0.001
Lifetime	33.8% (32.2%, 35.5%)	31.6% (29.9%, 33.4%)	68% (61.7%, 73.8%)	38.5% (29%, 48.9%)	< 0.001	38.3% (31.3%, 45.8%)	56.1% (49.8%, 62.2%)	56% (51.7%, 60.2%)	48.1% (43.9%, 52.2%)	27.4% (24.1%, 31.1%)	13% (10.7%, 15.7%)	5.6% (4.1%, 7.7%)	< 0.001
*Bitten*													
Past year	15.3% (14%, 16.6%)	13.4% (12.1%, 14.8%)	42.4% (36.2%, 48.9%)	18% (11.3%, 27.2%)	< 0.001	26% (20%, 33.1%)	31% (25.7%, 36.9%)	26.9% (23.3%, 30.9%)	19.7% (16.6%, 23.2%)	7.9% (5.9%, 10.6%)	3.8% (2.5%, 5.7%)	1.8% (1%, 3.3%)	< 0.001
Lifetime	26.6% (25.1%, 28.1%)	23.7% (22.2%, 25.3%)	63.5% (57.1%, 69.6%)	38% (28.6%, 48.3%)	< 0.001	30.3% (23.9%, 37.5%)	41.3% (35.4%, 47.4%)	44.1% (39.9%, 48.3%)	36.5% (32.6%, 40.6%)	21.4% (18.3%, 24.8%)	11.6% (9.4%, 14.2%)	6.3% (4.7%, 8.4%)	< 0.001
*Face slapped*													
Past year	2.9% (2.4%, 3.6%)	2.4% (1.8%, 3.1%)	9.2% (6.1%, 13.5%)	3.9% (1.5%, 9.9%)	< 0.001	5.6% (3%, 10%)	7.6% (4.9%, 11.8%)	6% (4.3%, 8.3%)	2.7% (1.7%, 4.4%)	0.5% (0.2%, 1.5%)	0.4% (0.1%, 1.1%)	0.5% (0.2%, 1.5%)	< 0.001
Lifetime	5.9% (5.1%, 6.8%)	4.8% (4%, 5.7%)	19.6% (15%, 25.1%)	8.3% (4.2%, 15.6%)	< 0.001	10.5% (6.7%, 15.9%)	13.3% (9.6%, 18%)	12% (9.6%, 15.1%)	5.5% (3.9%, 7.7%)	2.5% (1.6%, 4.1%)	1.5% (0.8%, 2.8%)	0.7% (0.2%, 1.8%)	< 0.001
*Genital area slapped*													
Past year	8.7% (7.7%, 9.8%)	7.7% (6.7%, 8.8%)	22.5% (17.4%, 28.4%)	5.6% (2.8%, 11.1%)	< 0.001	8.2% (4.8%, 13.4%)	18.7% (14.4%, 24.1%)	16.9% (13.9%, 20.4%)	11.7% (9.3%, 14.5%)	5.5% (3.9%, 7.7%)	2% (1.2%, 3.3%)	0.4% (0.1%, 1.2%)	< 0.001
Lifetime	14% (12.9%, 15.3%)	12.5% (11.3%, 13.8%)	34.1% (28.3%, 40.3%)	15.3% (9.5%, 23.9%)	< 0.001	10.1% (6.4%, 15.5%)	24.8% (19.9%, 30.4%)	23.6% (20.2%, 27.3%)	18.5% (15.5%, 21.8%)	12.5% (10.2%, 15.3%)	6.3% (4.8%, 8.3%)	3.2% (2.1%, 4.9%)	< 0.001
*Spanked lightly*													
Past year	28% (26.4%, 29.6%)	26.2% (24.5%, 27.9%)	57.8% (51.4%, 64.1%)	21.3% (13.9%, 31.3%)	< 0.001	35.6% (28.7%, 43.1%)	52.2% (46%, 58.3%)	51.6% (47.3%, 55.8%)	37.4% (33.4%, 41.5%)	19.4% (16.2%, 22.9%)	6.8% (5.1%, 9%)	3.3% (2.1%, 5.3%)	< 0.001
Lifetime	43.5% (41.8%, 45.3%)	41.7% (39.8%, 43.5%)	75.1% (68.9%, 80.5%)	37.4% (28.1%, 47.7%)	< 0.001	39.5% (32.4%, 47%)	60.7% (54.4%, 66.6%)	67% (62.8%, 70.9%)	59.4% (55.2%, 63.4%)	44% (40%, 48.1%)	23.9% (20.9%, 27.2%)	10.6% (8.4%, 13.3%)	< 0.001
*Spanked hard enough to leave a mark*													
Past year	9.8% (8.8%, 11%)	8.1% (7.1%, 9.3%)	35.4% (29.5%, 41.9%)	12.6% (6.9%, 22%)	< 0.001	19.2% (13.8%, 25.9%)	25.2% (20.3%, 30.8%)	17.9% (15%, 21.3%)	11.5% (9.1%, 14.4%)	3% (1.9%, 4.5%)	1.1% (0.5%, 2.4%)	0.6% (0.2%, 1.9%)	< 0.001
Lifetime	16.4% (15.1%, 17.7%)	13.6% (12.3%, 14.9%)	54.4% (47.9%, 60.6%)	25.1% (16.9%, 35.4%)	< 0.001	25% (19%, 32.1%)	34% (28.5%, 40%)	29.1% (25.4%, 32.9%)	19% (16%, 22.3%)	10% (7.9%, 12.5%)	4.8% (3.4%, 6.7%)	2.1% (1.2%, 3.7%)	< 0.001
*Choked*													
Past year	10.9% (9.8%, 12.1%)	8.7% (7.6%, 9.9%)	42% (35.8%, 48.4%)	11.9% (6.6%, 20.7%)	< 0.001	28.5% (22.2%, 35.7%)	32.7% (27.3%, 38.6%)	18.7% (15.8%, 22.1%)	9.8% (7.6%, 12.4%)	1.9% (1.1%, 3.3%)	0.4% (0.1%, 1.1%)	0.5% (0.2%, 1.4%)	< 0.001
Lifetime	15.8% (14.5%, 17.1%)	13.0% (11.7%, 14.3%)	55.0% (48.6%, 61.3%)	17.7% (11%, 27%)	< 0.001	31.9% (25.4%, 39.3%)	40.6% (34.8%, 46.7%)	29.8% (26.2%, 33.7%)	17.2% (14.3%, 20.6%)	5.1% (3.7%, 7.1%)	1.0% (0.5%, 2%)	1.0% (0.5%, 2.1%)	< 0.001
*Punched*													
Past year	0.7% (0.5%, 1.1%)	0.5% (0.3%, 0.8%)	3% (1.3%, 6.6%)	0.7% (0.1%, 5.2%)	< 0.001	1.6% (0.6%, 4.3%)	0.3% (0%, 1.9%)	1.7% (0.9%, 3.2%)	1.2% (0.6%, 2.5%)	0% (0%, 0%)	0% (0%, 0%)	0.6% (0.2%, 1.6%)	0.001
Lifetime	1.6% (1.3%, 2.2%)	1.4% (1.1%, 2%)	4% (2.1%, 7.5%)	3.1% (1%, 9.5%)	0.007	2.1% (0.9%, 5.2%)	1.5% (0.5%, 4.1%)	3.5% (2.2%, 5.4%)	2.7% (1.6%, 4.5%)	0.6% (0.2%, 1.6%)	0.3% (0.1%, 1%)	1.1% (0.5%, 2.3%)	< 0.001
*Called names*													
Past year	5.7% (5%, 6.6%)	4.8% (4%, 5.7%)	19.1% (14.5%, 24.7%)	7.4% (3.4%, 15.4%)	< 0.001	8.4% (5.1%, 13.4%)	14.5% (10.7%, 19.4%)	10.7% (8.4%, 13.6%)	6.1% (4.4%, 8.4%)	2.7% (1.7%, 4.3%)	1.5% (0.8%, 2.8%)	0.3% (0.1%, 1.1%)	< 0.001
Lifetime	11.5% (10.5%, 12.7%)	10% (9%, 11.2%)	32% (26.3%, 38.2%)	15.2% (9.2%, 24.2%)	< 0.001	12.1% (8.1%, 17.6%)	20.9% (16.3%, 26.3%)	18.5% (15.5%, 21.9%)	12.9% (10.4%, 15.9%)	9.7% (7.6%, 12.3%)	5.4% (4%, 7.3%)	4.1% (2.8%, 6%)	< 0.001
*Smothered during sexual activities*													
Past year	2.3% (1.8%, 2.9%)	1.7% (1.2%, 2.2%)	9% (5.8%, 13.6%)	3.3% (1%, 10.2%)	< 0.001	7.5% (4.4%, 12.6%)	4% (2.3%, 7.1%)	4.6% (3.1%, 6.8%)	2.2% (1.3%, 3.7%)	0.4% (0.1%, 1.1%)	0.4% (0.1%, 1.1%)	0.3% (0.1%, 1.2%)	< 0.001
Lifetime	3.9% (3.2%, 4.6%)	3% (2.4%, 3.8%)	13.5% (9.6%, 18.6%)	5.6% (2.3%, 13.2%)	< 0.001	8% (4.8%, 13.2%)	5.7% (3.5%, 9.1%)	7.9% (5.9%, 10.5%)	4.9% (3.4%, 7.1%)	0.9% (0.4%, 1.7%)	1% (0.5%, 2.1%)	1.4% (0.7%, 2.8%)	< 0.001
*Any of these with permission or consent*													
Past year	34.6% (32.9%, 36.3%)	32.5% (30.8%, 34.3%)	69.5% (63.3%, 75%)	26.7% (18.6%, 36.9%)	< 0.001	41.7% (34.6%, 49.2%)	59.2% (53%, 65.2%)	59.6% (55.4%, 63.7%)	47.2% (43.1%, 51.4%)	25.1% (21.7%, 28.9%)	11.8% (9.6%, 14.5%)	8% (6.1%, 10.4%)	< 0.001
Lifetime	53.8% (52.1%, 55.5%)	51.8% (50%, 53.6%)	83.6% (77.9%, 88.1%)	56.1% (45.6%, 66%)	< 0.001	46.9% (39.6%, 54.4%)	70.1% (64%, 75.5%)	75.2% (71.2%, 78.8%)	70.6% (66.6%, 74.3%)	56% (51.9%, 59.9%)	33.9% (30.5%, 37.4%)	23.1% (20%, 26.4%)	< 0.001

In terms of sexual orientation identity, each of these lifetime behaviors was significantly more likely to be reported by sexual minority women (bisexual/pansexual and lesbian women) as compared to heterosexual women. As one example, hair pulling was reported by 57.9% of bisexual/pansexual women, 43.1% of lesbian women, and 24.9% of heterosexual women (*p* < 0.001). As a second example, 31.0% of bisexual/pansexual women, 20.9% of lesbian women, and 7.5% of heterosexual women reported having ever choked a partner (*p* < 0.001). As a third example, 15.0% of bisexual/pansexual women, 17.3% of lesbian women, and 7.9% of heterosexual women reported having ever slapped a partner on their genitals (*p* < 0.001). There were no significant differences by sexual orientation identity in terms of having ever punched a partner, which was reported by 1% of women in the past year (95% CI: 0.7%, 1.4%) and 1.8% of women ever in their lifetime (95% CI: 1.4%, 2.3%).

In terms of age, each of the lifetime rough sex behaviors assessed was significantly more likely to be reported by younger women as compared with older women (*p* < 0.001 for each behavior). For example, while 2.1% or fewer of women ages 50 and older reported having ever slapped a partner on their face, this was reported by 6.0% of 18–24-year-old women, 11.3% of 25–29-year-old women, 10.9% of 30–39-year-old women, and 6.0% of 40–49-year-old women (*p* < 0.001). Also, while having ever choked a partner was reported by 2.9% of women ages 50–59, and 0.6% of women aged 60 and older, we found that 19.1% of 18–24-year-old women, 24.0% of 25–29-year-old women, 17.5% of 30–39-year-old women, and 9.8% of 40–49-year-old women (*p* < 0.001) had ever choked a partner.

### Women’s Reports of Receiving Rough Sex Behaviors, with Permission or Consent

As shown in Table 2, 53.8% of women (95% CI: 52.1%, 55.5%) reported that a partner had ever done at least one of the assessed behaviors to them, with permission or consent. Also, 34.6% of women (95% CI: 32.9%, 36.3%) reported that this had occurred during the past year. Of the sexual practices we assessed, the five that were most often reported to have ever been done to them with permission or consent were having: been spanked lightly (43.5%), had their hair pulled (33.8%), been bitten (26.6%), been spanked hard enough to leave a mark (16.4%), and been choked (15.8%).

In terms of sexual orientation identity, there were significant differences observed for most of the sexual behaviors assessed, with the general pattern being that bisexual/pansexual women were more likely than heterosexual and lesbian women to report having been on the receiving end of each of the rough sex practices, both ever and in the past year. As one example, having been spanked lightly with permission or consent was reported by 75.1% of bisexual/pansexual women, 41.7% of heterosexual women, and 37.4% of lesbian women (*p* < 0.001). As a second example, 54.4% of bisexual/pansexual women, 25.1% of lesbian women, and 13.6% of heterosexual women reported having been spanked hard enough to leave a mark with permission or consent (*p* < 0.001). As a third example, 55.0% of bisexual/pansexual women, 17.7% of lesbian women, and 13.0% of heterosexual women reported having been choked during sexual activities, with permission or consent (*p* < 0.001).

In terms of age, each of the behaviors we assessed was significantly more likely to be reported by younger women as compared with older women, both ever and in the prior year. As a general pattern, more women between ages 18 and 39 reported having received certain rough sex practices. As one example, having had one’s face slapped with permission or consent was rare for women ages 40 and older (5.5% for 40–49-year-old women, 2.5% for 50–59-year-old women, 1.5% for 60–69-year-old women, and 0.7% for 70 + year-old women). However, we found that more than 10% of women ages 18 to 39 reported that a partner had ever slapped their face with permission or consent (*p* < 0.001). As a second example, while 5.1% of women in their 50 s and 1.0% of women in their 60 s and above reported having ever been choked with permission or consent, having been consensually choked was commonly reported by younger women: 31.9% of 18–24-year-old women, 40.6% of 25–29-year-old women, 29.8% of 30–39-year-old women, and 17.2% of 40–49-year-old women, demonstrating an age cohort effect (*p* < 0.001). As a third example, few women in their 60 s (6.3%) or those ages 70 + (3.2%) reported that a partner had ever consensually slapped their genitals during sexual activities, though this was relatively common among younger adult women. Specifically, 10.1% of 18–24-year-old women, 24.8% of 25–29 year-old women, 23.6% of women in their 30 s, 18.5% of women in their 40 s, and 12.5% of women in their 50 s (*p* < 0.001) reported that a partner had ever slapped their genitals with permission or consent.

### Men’s Reports of Enacting Rough Sex Behaviors

As shown in Table 3, 60.8% of men (95% CI: 59.1%, 62.5%) reported having ever enacted one or more of the assessed sexual practices on a partner. Also, 39.2% of men (95% CI: 37.5%, 41.0%) had enacted one or more of these behaviors in the last year. Each of these is higher than the percentages of women who reported having enacted one of these rough sex behaviors on a partner, whether in the past year or ever in their lives. The five most commonly reported lifetime behaviors that men reported having ever done to a partner were: spanking them lightly (53.0%), pulling their hair (39.6%), biting them (33.1%), slapping their genitals (18.2%), and choking their partner(s) (15.8%).

**Table 3:** Men's past year and lifetime rough sex experiences by sexual orientation identity and age

	Overall	Sexual Orientation Identity				Age							
		Heterosexual	Bisexual/Pansexual	Lesbian or Gay	*p*-value	18–24	25–29	30–39	40–49	50–59	60–69	70 +	*p*-value
	*N* = 4346	*N* = 3933	*N* = 100	*N* = 187		*N* = 425	*N* = 443	*N* = 689	*N* = 785	*N* = 743	*N* = 681	*N* = 580	
*Participant did this to a partner*													
*Pulled their hair*													
Past year	22.1% (20.6%, 23.6%)	22.1% (20.5%, 23.8%)	25.1% (17.7%, 34.4%)	15.5% (11.3%, 20.8%)	0.056	20.3% (14.8%, 27.3%)	35.1% (28.9%, 41.8%)	37% (32.8%, 41.4%)	30.4% (26.9%, 34.2%)	18.6% (15.6%, 22.1%)	9.1% (7.1%, 11.6%)	4.0% (2.6%, 6.1%)	< 0.001
Lifetime	39.6% (37.9%, 41.3%)	39.5% (37.6%, 41.3%)	46.6% (36.7%, 56.7%)	37.8% (31.3%, 44.7%)	0.313	31.1% (24.4%, 38.7%)	46.1% (39.4%, 52.8%)	57.2% (52.8%, 61.5%)	52.3% (48.3%, 56.4%)	44.7% (40.8%, 48.7%)	24.3% (21.2%, 27.8%)	13.7% (11.2%, 16.6%)	< 0.001
*Bit them*													
Past year	17.3% (16%, 18.8%)	16.9% (15.4%, 18.4%)	25.4% (17.7%, 35%)	19% (14.2%, 25.1%)	0.067	14.5% (9.9%, 20.8%)	32.1% (26.2%, 38.6%)	29% (25.2%, 33.2%)	24.0% (20.6%, 27.7%)	13.3% (10.7%, 16.3%)	6.6% (4.9%, 8.7%)	2.9% (1.8%, 4.7%)	< 0.001
Lifetime	33.1% (31.4%, 34.8%)	32.6% (30.9%, 34.4%)	41.9% (32.4%, 52.1%)	38.2% (31.8%, 45.1%)	0.045	21.6% (15.9%, 28.6%)	40.1% (33.7%, 46.8%)	48.8% (44.4%, 53.2%)	41.8% (37.8%, 45.8%)	36.3% (32.6%, 40.2%)	21.1% (18.1%, 24.4%)	15.4% (12.7%, 18.5%)	< 0.001
*Slapped their face*													
Past year	4.1% (3.4%, 4.9%)	3.7% (3%, 4.6%)	7.6% (3.9%, 14%)	9.9% (6.2%, 15.3%)	< 0.001	6.0% (3.2%, 11%)	9.1% (5.9%, 13.7%)	8.2% (6%, 11%)	4.8% (3.4%, 6.9%)	1.1% (0.6%, 2.1%)	0.8% (0.3%, 1.9%)	0.7% (0.2%, 2.4%)	< 0.001
Lifetime	8.9% (7.9%, 10%)	8.2% (7.1%, 9.3%)	14.7% (9.4%, 22.3%)	20.3% (15%, 26.7%)	< 0.001	9.8% (6%, 15.6%)	15.7% (11.4%, 21.2%)	17.1% (14%, 20.7%)	10.4% (8.2%, 13%)	5.1% (3.6%, 7.1%)	2.5% (1.5%, 4%)	3.4% (2.1%, 5.6%)	< 0.001
*Slapped their genital area*													
Past year	9.8% (8.8%, 11%)	9.8% (8.7%, 11%)	10.8% (6.1%, 18.5%)	10% (6.7%, 14.8%)	0.934	6.9% (3.9%, 12.1%)	15% (10.8%, 20.5%)	15.4% (12.3%, 19%)	13.9% (11.3%, 17%)	8.9% (6.8%, 11.6%)	4.4% (3.1%, 6.3%)	3.4% (2.1%, 5.7%)	< 0.001
Lifetime	18.3% (16.9%, 19.7%)	18.1% (16.7%, 19.6%)	24.2% (17%, 33.2%)	20.4% (15.5%, 26.3%)	0.2	11.5% (7.4%, 17.5%)	19.7% (14.8%, 25.7%)	25.4% (21.7%, 29.5%)	24.7% (21.4%, 28.4%)	19.8% (16.8%, 23.2%)	11.5% (9.3%, 14.1%)	10.8% (8.4%, 13.7%)	< 0.001
*Spanked them lightly*													
Past year	33.1% (31.4%, 34.9%)	33.1% (31.3%, 34.9%)	34.9% (25.9%, 45.1%)	32.3% (26.3%, 38.9%)	0.902	29.2% (22.7%, 36.8%)	49.7% (42.9%, 56.4%)	51.2% (46.7%, 55.6%)	47% (43%, 51.1%)	30.4% (26.7%, 34.3%)	15.5% (12.8%, 18.6%)	7.0% (5%, 9.6%)	< 0.001
Lifetime	53.0% (51.3%, 54.8%)	52.8% (50.9%, 54.7%)	61.8% (51.4%, 71.2%)	55.5% (48.4%, 62.3%)	0.176	36.2% (29.1%, 44%)	58.1% (51.2%, 64.7%)	69.1% (64.8%, 73.1%)	68.3% (64.4%, 71.9%)	59.6% (55.7%, 63.4%)	42% (38.2%, 45.8%)	25.4% (22.1%, 29%)	< 0.001
*Spanked hard enough to leave a mark*													
Past year	11% (9.9%, 12.2%)	10.6% (9.4%, 11.9%)	18.4% (11.5%, 28.2%)	12.3% (8.5%, 17.4%)	0.045	11.8% (7.7%, 17.7%)	22.2% (17.3%, 28.2%)	19.6% (16.3%, 23.4%)	13.8% (11.2%, 16.8%)	6.5% (4.8%, 8.8%)	4% (2.5%, 6.2%)	1.6% (0.8%, 3.4%)	< 0.001
Lifetime	19.5% (18.1%, 21%)	19.0% (17.5%, 20.5%)	33.5% (24.2%, 43.1%)	19.7% (15%, 25.4%)	0.002	14.4% (9.8%, 20.7%)	30.5% (24.7%, 36.9%)	31.4% (27.4%, 35.6%)	26.1% (22.7%, 29.8%)	17.2% (14.4%, 20.3%)	10.3% (8%, 13%)	5.5% (3.9%, 7.7%)	< 0.001
*Choked them*													
Past year	9.7% (8.7%, 10.9%)	9.3% (8.2%, 10.6%)	15.1% (9.1%, 24.2%)	14.4% (10.1%, 20.1%)	0.016	12.8% (8.5%, 18.9%)	26.2% (20.7%, 32.5%)	19.8% (16.6%, 23.6%)	9.2% (7.2%, 11.7%)	3.2% (2.1%, 4.8%)	2.1% (1.2%, 3.6%)	1.1% (0.5%, 2.7%)	< 0.001
Lifetime	15.8% (14.5%, 17.2%)	15.1% (13.8%, 16.6%)	27.9% (19.6%, 37.9%)	21.7% (16.6%, 27.9%)	< 0.001	20.4% (14.9%, 27.4%)	31.5% (25.6%, 38%)	31.9% (27.9%, 36.1%)	17.3% (14.5%, 20.5%)	9.1% (7.2%, 11.5%)	3.7% (2.5%, 5.6%)	2.2% (1.2%, 4%)	< 0.001
*Punched them*													
Past year	1% (0.6%, 1.4%)	0.9% (0.6%, 1.4%)	0.6% (0.1%, 4.4%)	2.9% (1.3%, 6.1%)	0.013	1.7% (0.5%, 5.4%)	0.5% (0.1%, 2%)	3.1% (1.7%, 5.4%)	0.6% (0.2%, 1.6%)	0% (0%, 0.1%)	0.3% (0.1%, 1.2%)	0.8% (0.3%, 2.5%)	< 0.001
Lifetime	1.9% (1.5%, 2.5%)	1.7% (1.3%, 2.4%)	2.4% (0.9%, 6.4%)	5.5% (3.2%, 9.2%)	< 0.001	1.7% (0.5%, 5.4%)	1.3% (0.4%, 4%)	4.8% (3.2%, 7.3%)	2.3% (1.4%, 3.8%)	0.5% (0.2%, 1.2%)	1.1% (0.5%, 2.1%)	1.5% (0.6%, 3.3%)	< 0.001
*Called them names*													
Past year	6.7% (5.8%, 7.6%)	5.9% (5.1%, 6.9%)	12% (7.3%, 19%)	16.4% (11.8%, 22.3%)	< 0.001	5.4% (3%, 9.5%)	11.5% (7.9%, 16.4%)	11.3% (8.8%, 14.5%)	8.1% (6.1%, 10.5%)	5.5% (3.9%, 7.6%)	3.6% (2.4%, 5.4%)	1.5% (0.7%, 3%)	< 0.001
Lifetime	13.1% (12%, 14.3%)	12% (10.8%, 13.3%)	25.1% (17.5%, 34.6%)	27.2% (21.5%, 33.8%)	< 0.001	7.3% (4.3%, 12.1%)	17.2% (12.7%, 22.9%)	17.6% (14.5%, 21.2%)	16% (13.3%, 19.1%)	13.9% (11.3%, 16.9%)	10.6% (8.5%, 13.2%)	6.6% (4.9%, 9%)	< 0.001
*Smothered them during sexual activities*													
Past year	2.6% (2%, 3.3%)	2.4% (1.8%, 3.1%)	0.6% (0.1%, 2.6%)	4.9% (2.7%, 8.8%)	0.016	3.7% (1.7%, 8.2%)	7.2% (4.5%, 11.3%)	5.3% (3.6%, 7.8%)	1.9% (1.1%, 3.3%)	0.5% (0.2%, 1.4%)	0.6% (0.2%, 1.5%)	0.9% (0.3%, 2.5%)	< 0.001
Lifetime	4.4% (3.7%, 5.3%)	4.1% (3.4%, 5%)	6.4% (3.1%, 12.7%)	6.9% (4.2%, 11.2%)	0.088	5.5% (2.8%, 10.5%)	8.7% (5.7%, 13.1%)	9.1% (6.8%, 12.1%)	5.4% (3.9%, 7.5%)	1.6% (0.9%, 2.8%)	1% (0.5%, 2.1%)	1.1% (0.5%, 2.6%)	< 0.001
*Did any of these to a partner*													
Past year	39.2% (37.5%, 41.0%)	39.1% (37.2%, 40.9%)	41.6% (32.1%, 51.8%)	40% (33.5%, 46.9%)	0.846	34.4% (27.4%, 42.1%)	52.8% (46%, 59.5%)	59.7% (55.3%, 64%)	53.7% (49.7%, 57.7%)	37.3% (33.5%, 41.3%)	21.3% (18.3%, 24.7%)	12% (9.4%, 15.1%)	< 0.001
Lifetime	60.8% (59.1%, 62.5%)	60.4% (58.6%, 62.2%)	68.9% (58.6%, 77.7%)	67.2% (60.2%, 73.5%)	0.054	41.9% (34.5%, 49.7%)	60.7% (53.9%, 67.2%)	76.1% (72%, 79.7%)	75.2% (71.6%, 78.5%)	69.3% (65.6%, 72.8%)	50.4% (46.6%, 54.2%)	37.5% (33.8%, 41.4%)	< 0.001
*Done to participant with consent*													
*Hair was pulled*													
Past year	8.7% (7.7%, 9.8%)	8% (7%, 9.2%)	15.4% (9.6%, 23.7%)	13.1% (9%, 18.7%)	0.002	7.8% (4.6%, 12.9%)	20.9% (15.9%, 26.9%)	15.3% (12.3%, 18.7%)	9.2% (7%, 11.8%)	6.6% (4.8%, 9%)	2.7% (1.7%, 4.2%)	1.3% (0.5%, 2.9%)	< 0.001
Lifetime	15.8% (14.5%, 17.1%)	14.7% (13.4%, 16.1%)	26.5% (19%, 35.6%)	27.7% (22%, 34.3%)	< 0.001	11.1% (7.2%, 16.8%)	25.6% (20.1%, 31.9%)	26.1% (22.4%, 30.1%)	17.9% (15%, 21.3%)	16% (13.3%, 19.1%)	8.1% (6.2%, 10.4%)	5.2% (3.7%, 7.2%)	< 0.001
*Bitten*													
Past year	15% (13.7%, 16.4%)	14.5% (13.1%, 15.9%)	25.8% (17.8%, 35.7%)	18.7% (13.9%, 24.8%)	0.003	14.8% (10.1%, 21.2%)	29% (23.2%, 35.5%)	23.8% (20.2%, 27.8%)	19.6% (16.5%, 23.1%)	12.7% (10.3%, 15.7%)	5% (3.6%, 6.9%)	2.4% (1.4%, 4.1%)	< 0.001
Lifetime	29.8% (28.2%, 31.4%)	29.3% (27.6%, 31.1%)	43.8% (34%, 54%)	32.9% (26.9%, 39.5%)	0.005	19.9% (14.4%, 26.8%)	36.4% (30.2%, 43.1%)	43% (38.7%, 47.4%)	36.4% (32.6%, 40.4%)	35.8% (32.1%, 39.6%)	19% (16.2%, 22.2%)	11.8% (9.5%, 14.5%)	< 0.001
*Face slapped*													
Past year	2.7% (2.1%, 3.3%)	2.4% (1.9%, 3.1%)	6.0% (2.9%, 12.1%)	6.1% (3.7%, 9.9%)	< 0.001	3.6% (1.6%, 8%)	3.9% (2.2%, 7%)	6.5% (4.7%, 9.1%)	2.8% (1.8%, 4.5%)	0.9% (0.4%, 2.1%)	0.8% (0.3%, 2%)	0.6% (0.2%, 2.4%)	< 0.001
Lifetime	5.6% (4.9%, 6.5%)	4.9% (4.1%, 5.8%)	11.7% (6.8%, 19.3%)	18.0% (13.2%, 24.1%)	< 0.001	5.5% (2.9%, 10.3%)	8.2% (5.2%, 12.6%)	10.9% (8.5%, 13.9%)	7.8% (5.8%, 10.3%)	2.8% (1.8%, 4.4%)	2.3% (1.4%, 3.7%)	2.1% (1.2%, 3.7%)	< 0.001
*Genital area slapped*													
Past year	4.8% (4%, 5.6%)	4.3% (3.6%, 5.2%)	6.1% (2.9%, 12.4%)	11.7% (8%, 16.7%)	< 0.001	4.0% (1.8%, 8.6%)	7.3% (4.5%, 11.7%)	8.1% (6%, 10.9%)	5.4% (3.8%, 7.6%)	4.6% (3.2%, 6.7%)	2.5% (1.6%, 4%)	1.3% (0.6%, 2.9%)	< 0.001
Lifetime	9.5% (8.5%, 10.6%)	8.6% (7.6%, 9.8%)	18.5% (12.1%, 27.1%)	20.7% (15.8%, 26.8%)	< 0.001	6.4% (3.5%, 11.4%)	10.7% (7.1%, 15.7%)	13.2% (10.5%, 16.5%)	11.9% (9.5%, 15%)	9.8% (7.7%, 12.4%)	6.7% (5.1%, 8.9%)	5.6% (4.1%, 7.7%)	0.001
*Spanked lightly*													
Past year	14.6% (13.4%, 15.9%)	13.4% (12.1%, 14.7%)	26% (17.8%, 36.2%)	29.7% (23.9%, 36.3%)	< 0.001	12.4% (8.3%, 18.1%)	21.2% (16.4%, 27%)	23.9% (20.5%, 27.8%)	21% (17.8%, 24.6%)	13.2% (10.7%, 16.1%)	6.5% (4.7%, 8.9%)	2.6% (1.4%, 4.7%)	< 0.001
Lifetime	25.8% (24.3%, 27.4%)	23.8% (22.2%, 25.4%)	49.8% (39.6%, 60%)	52.1% (45.1%, 59%)	< 0.001	14.6% (10%, 20.6%)	28.2% (22.6%, 34.5%)	35.4% (31.4%, 39.7%)	35.3% (31.5%, 39.3%)	30.9% (27.3%, 34.6%)	18.1% (15.3%, 21.3%)	9.8% (7.6%, 12.5%)	< 0.001
*Spanked hard enough to leave a mark*													
Past year	4.4% (3.7%, 5.3%)	3.8% (3.1%, 4.6%)	10.7% (5.9%, 18.6%)	11.7% (8%, 16.9%)	< 0.001	3.5% (1.6%, 7.5%)	9.8% (6.5%, 14.4%)	8.1% (6%, 10.9%)	4.7% (3.3%, 6.7%)	2.9% (1.8%, 4.6%)	1.3% (0.5%, 2.9%)	2.0% (0.9%, 4%)	< 0.001
Lifetime	9.0% (8.1%, 10.1%)	7.7% (6.8%, 8.8%)	21.9% (14.7%, 31.2%)	24.0% (18.8%, 30.2%)	< 0.001	5.8% (3.1%, 10.5%)	14% (10%, 19.2%)	16.5% (13.5%, 20%)	11.1% (8.9%, 13.8%)	7.9% (6.1%, 10.2%)	3.4% (2.2%, 5.1%)	3.7% (2.3%, 5.8%)	< 0.001
*Choked*													
Past year	4.3% (3.6%, 5.2%)	3.8% (3.1%, 4.7%)	9.5% (4.9%, 17.8%)	11.9% (8.1%, 17.3%)	< 0.001	5.3% (2.8%, 9.7%)	13.2% (9.1%, 18.7%)	8.8% (6.6%, 11.6%)	3.1% (2%, 4.8%)	1.2% (0.6%, 2.3%)	0.6% (0.3%, 1.4%)	1.3% (0.5%, 3.1%)	< 0.001
Lifetime	7.2% (6.3%, 8.2%)	6.3% (5.4%, 7.4%)	18.0% (11.3%, 27.4%)	21.3% (16.1%, 27.6%)	< 0.001	8.1% (4.9%, 13.2%)	17.5% (12.9%, 23.5%)	14.4% (11.6%, 17.7%)	7.6% (5.7%, 10%)	2.7% (1.8%, 4.2%)	1.4% (0.8%, 2.5%)	1.9% (0.9%, 3.7%)	< 0.001
*Punched*													
Past year	1.1% (0.7%, 1.5%)	1.0% (0.7%, 1.5%)	0.6% (0.1%, 4.4%)	2.2% (0.9%, 4.9%)	0.178	1.0% (0.2%, 4.6%)	1.8% (0.7%, 4.5%)	3.1% (1.8%, 5.1%)	0.8% (0.4%, 1.7%)	0.1% (0%, 0.5%)	0.2% (0%, 1.3%)	0.8% (0.3%, 2.5%)	< 0.001
Lifetime	2.2% (1.7%, 2.8%)	2% (1.5%, 2.6%)	4.4% (1.8%, 10.9%)	4.6% (2.6%, 8.1%)	0.011	3% (1.2%, 7.4%)	2.4% (1%, 5.8%)	4.7% (3.1%, 7%)	2.2% (1.3%, 3.5%)	0.9% (0.5%, 1.7%)	0.6% (0.3%, 1.4%)	1.7% (0.9%, 3.4%)	0.001
*Called names*													
Past year	3.5% (2.9%, 4.2%)	2.4% (1.9%, 3.1%)	11.6% (6.7%, 19.2%)	18.5% (13.5%, 24.7%)	< 0.001	2.9% (1.2%, 6.6%)	7.1% (4.4%, 11.3%)	6.1% (4.4%, 8.6%)	3.7% (2.5%, 5.5%)	2.6% (1.6%, 4.1%)	1.7% (0.9%, 3%)	1.0% (0.4%, 2.5%)	< 0.001
Lifetime	7.4% (6.5%, 8.4%)	5.7% (4.9%, 6.7%)	25.9% (18.2%, 35.5%)	29.5% (23.5%, 36.2%)	< 0.001	6.4% (3.5%, 11.5%)	11.4% (7.7%, 16.4%)	9.9% (7.6%, 12.8%)	8.0% (6.2%, 10.3%)	7.8% (6%, 10%)	5.3% (3.9%, 7.1%)	3.3% (2.1%, 5%)	0.001
*Smothered during sexual activities*													
Past year	1.2% (0.9%, 1.7%)	1% (0.7%, 1.6%)	1.7% (0.5%, 5.2%)	3.7% (1.9%, 7%)	0.001	2.3% (0.8%, 6.2%)	0.6% (0.2%, 2%)	4.2% (2.6%, 6.5%)	1.0% (0.5%, 2.1%)	0% (0%, 0.1%)	0.2% (0%, 1.3%)	0.6% (0.1%, 2.4%)	< 0.001
Lifetime	2.5% (2%, 3.1%)	2.1% (1.6%, 2.8%)	5.5% (2.4%, 11.9%)	7.1% (4.4%, 11.2%)	< 0.001	3.8% (1.7%, 8.5%)	1.7% (0.7%, 4%)	6.6% (4.6%, 9.2%)	2.6% (1.7%, 4.1%)	1% (0.5%, 2.1%)	0.6% (0.2%, 1.5%)	1.4% (0.6%, 3%)	< 0.001
*Any of these with permission or consent*													
Past year	26.5% (25.0%, 28.1%)	25.3% (23.7%, 27.1%)	39.1% (29.6%, 49.6%)	42% (35.4%, 48.9%)	< 0.001	24.2% (18.2%, 31.3%)	41.3% (34.9%, 48.1%)	41.3% (37.1%, 45.7%)	34.9% (31.1%, 38.9%)	23.2% (20%, 26.7%)	12.3% (10%, 15.1%)	8.9% (6.8%, 11.6%)	< 0.001
Lifetime	45.8% (44.0%, 47.5%)	44.2% (42.4%, 46.1%)	69.1% (59.1%, 77.6%)	67.8% (60.7%, 74.1%)	< 0.001	32.6% (25.8%, 40.2%)	49.5% (42.7%, 56.2%)	58.6% (54.2%, 62.9%)	56.2% (52.2%, 60.2%)	51.9% (47.9%, 55.8%)	35.3% (31.8%, 39%)	27% (23.7%, 30.6%)	< 0.001

In terms of sexual orientation identity, there were significant differences observed for several of the sexual practices assessed. As one example, 20.3% of gay men, 14.7% of bisexual/pansexual men, and 8.2% of heterosexual men (*p* < 0.001) reported that they had ever slapped a partner’s face. As a second example, 33.5% of bisexual/pansexual men, 19.7% of gay men, and 19.0% of heterosexual men (*p* < 0.001) reported that they had ever spanked a partner hard enough to leave a mark. As a third example, 27.9% of bisexual/pansexual men, 21.7% of gay men, and 15.1% of heterosexual men (*p* < 0.001) reported that they had ever choked a partner during sexual activities. There were no significant differences observed for sexual orientation identity in relation to: past year or lifetime hair pulling, genital slapping, or light spanking, as well as past year biting.

In terms of age, each of the behaviors we assessed was significantly more likely to be reported by younger men as compared with older men (*p* < 0.001 for each behavior). While it was rare for men in their 50 s and beyond to report having ever slapped a partner’s face during sexual activities (5.1% of men in their 50 s, 2.5% in their 60 s, and 3.4% ages 70 +), it was more common among younger men (*p* < 0.001). That is, 9.8% of 18–24-year-old men, 15.7% of 25–29-year-old men, 17.1% of 30–39-year-old men, and 10.4% of 40–49-year-old men reported that they had ever slapped a partner’s face during sexual activities. Choking was also much less common among men ages 50 and older as compared with younger men. For example, having ever choked a partner was reported by 9.1% of men in their 50 s, 3.7% of men in their 60 s, and 2.2% of men ages 70 and older; however, it was reported by 20.4% by 18–24-year-old men, 31.5% of 25–29 year-old men, 31.9% of men ages 30–39, and 17.3% of men ages 40–49 (*p* < 0.001).

### Men’s Reports of Receiving Rough Sex Behaviors, with Permission or Consent

As shown in Table 3, 45.8% of men (95% CI: 44.0%, 47.5%) reported that a partner had ever performed at least one kind of the rough sex behaviors we assessed on them, with permission or consent. Also, 26.5% of men (95% CI: 25.0%, 28.1%) reported that this had occurred during the past year. Each of these is lower than the percentages of women who reported receiving one or more of these rough sex behaviors from a partner with permission or consent, whether in the past year or ever in their lives.

Of the sexual practices we assessed, the five that men most often reported had ever been done to them with permission or consent were having: been bitten (29.8%), been spanked lightly (25.8%), had their hair pulled (15.8%), had their genitals slapped (9.5%), and been spanked hard enough to leave a mark (9.0%).

In terms of sexual orientation identity, significant differences were observed for nearly all of the consensual sexual practices we assessed, with the exception of being punched with permission or consent in the prior year. As one example, 18.0% of gay men, 11.7% of bisexual/pansexual men, and 4.9% of heterosexual men reported having ever been slapped on their face with permission or consent (*p* < 0.001). Also, 20.7% of gay men, 18.5% of bisexual/pansexual men, and 8.6% of heterosexual men reported having ever had their genitals slapped with permission or consent (*p* < 0.001). Being choked with permission or consent was reported by 21.3% of gay men, 18.0% of bisexual/pansexual men, and 6.3% of heterosexual men.

In terms of age, we observed significant differences by age for all consensual sexual behaviors assessed. As one example, while 2.8% or fewer of the men ages 50 and older reported that a partner had ever slapped their face with consent, 5.5–10.9% of men between the ages of 18 and 49 reported that a partner had slapped their face. Similarly, while 2.7% or fewer of the men ages 50 and older reported having ever been choked with consent, this was reported by 7.6–17.6% of men in the age groups between 18 and 49.

### TGNB+ Participants’ Reports of Enacting Rough Sex Behaviors

Of the TGNB+ participants, 71.1% (95% CI: 60.3%, 80.0%) reported having done at least one of the rough sex practices assessed to a partner ever in their lives and 49.0% (95% CI: 38.6%, 59.5%) reported having done so in the past year (Table 4). The five most commonly reported lifetime behaviors they had done to a partner were: biting (62.5%), light spanking (55.4%), hair pulling (56.8%), spanking hard enough to leave a mark (37.4%), and choking (38.8%). See Table 4 for additional details. With the exception of past year hair pulling and past year biting, there were no significant differences in the percentage of TGNB+ participants who reported having done each sexual behavior to another person, in relation to their birth assigned sex. There were also no significant differences for TGNB+ participants’ reports of doing each of the ten sexual behaviors assessed ever in their lives, in relation to their birth assigned sex.

**Table 4:** Rough sex experiences reported by transgender, gender nonbinary, and other gender non-conforming participants

		Gender/Sex Assigned at Birth		
	Overall TGNB+	TGNB+/AFAB	TGNB+/AMAB	*p*-value
	*N* = 121	*N* = 83	*N* = 31	
*Participant did this to a partner*				
*Pulled their hair*				
Past year	34.9% (25.5%, 45.5%)	40.8% (28.9%, 54%)	13.7% (5.2%, 31.5%)	0.009
Lifetime	56.8% (46.1%, 66.9%)	56.1% (42.9%, 68.5%)	50.8% (31.8%, 69.5%)	0.647
*Bit them*				
Past year	38.9% (29.2%, 49.6%)	42.9% (30.8%, 55.9%)	18.3% (7.5%, 38.1%)	0.028
Lifetime	62.5% (51.6%, 72.2%)	61.6% (48.1%, 73.5%)	57.6% (37.7%, 75.3%)	0.731
*Slapped their face*				
Past year	17.8% (11%, 27.5%)	20% (11.7%, 32.2%)	4.1% (0.5%, 25.3%)	0.066
Lifetime	26.4% (18.1%, 36.9%)	29.1% (18.8%, 42.2%)	13.3% (4.7%, 32.5%)	0.113
*Slapped their genital* *area*				
Past year	16.5% (10%, 26.2%)	17.6% (9.7%, 29.8%)	5.5% (1.1%, 22.9%)	0.116
Lifetime	29% (20.4%, 39.5%)	27.1% (17.1%, 40.1%)	28.1% (14.2%, 48%)	0.922
*Spanked them lightly*				
Past year	36.4% (26.9%, 47.2%)	35.5% (24.2%, 48.8%)	27.8% (13.8%, 48%)	0.482
Lifetime	55.4% (44.6%, 65.7%)	53.1% (39.9%, 65.8%)	52.7% (33.4%, 71.1%)	0.973
*Spanked hard enough to leave a* mark				
Past year	23.3% (15.5%, 33.4%)	25.4% (15.9%, 38%)	11% (3.4%, 30.2%)	0.128
Lifetime	37.4% (27.9%, 48%)	37.9% (26.4%, 51%)	32.1% (17.1%, 52%)	0.599
*Choked them*				
Past year	23.8% (15.8%, 34.2%)	23.4% (14.2%, 36%)	11.5% (3.6%, 31.3%)	0.207
Lifetime	38.8% (29.1%, 49.6%)	35.3% (24%, 48.6%)	37.5% (20.9%, 57.6%)	0.848
*Punched them*				
Past year	10% (5%, 19.1%)	7% (2.5%, 18%)	8.2% (1.9%, 28.6%)	0.853
Lifetime	10.8% (5.6%, 19.8%)	8.1% (3.2%, 18.9%)	8.2% (1.9%, 28.6%)	0.993
*Called them names*				
Past year	20.2% (12.9%, 30.2%)	21.1% (12.4%, 33.4%)	10.6% (3%, 31.1%)	0.258
Lifetime	35.2% (25.8%, 45.9%)	35.3% (23.9%, 48.5%)	30.5% (15.9%, 50.5%)	0.666
*Smothered them during sexual* *activities*				
Past year	10.9% (5.5%, 20.4%)	7.3% (2.8%, 17.9%)	4.1% (0.5%, 25.7%)	0.595
Lifetime	19.6% (12.4%, 29.6%)	14.2% (7.3%, 25.6%)	19.2% (7.9%, 39.6%)	0.553
*Did any of these to a partner*				
Past year	49.0% (38.6%, 59.5%)	50.8% (37.9%, 63.5%)	35.9% (19.8%, 55.9%)	0.201
Lifetime	71.1% (60.3%, 80.0%)	71.6% (57.9%, 82.2%)	64.3% (43.5%, 80.9%)	0.519
*Done to participant with consent*				
*Hair was pulled*				
Past year	28.9% (20.2%, 39.4%)	32.3% (21.5%, 45.3%)	13.2% (4.6%, 32.6%)	0.063
Lifetime	56.8% (46.1%, 66.9%)	61.9% (48.5%, 73.8%)	42% (24.6%, 61.6%)	0.089
*Bitten*				
Past year	29.8% (21.1%, 40.3%)	31.4% (20.9%, 44.3%)	19.1% (7.9%, 39.4%)	0.239
Lifetime	58.7% (47.9%, 68.7%)	58.8% (45.4%, 71%)	56% (36.4%, 73.9%)	0.814
*Face slapped*				
Past year	14.6% (8.4%, 24.1%)	14.9% (7.9%, 26.4%)	4.1% (0.5%, 25.7%)	0.167
Lifetime	23.2% (15.5%, 33.2%)	23.5% (14.4%, 35.8%)	14.5% (5.5%, 33.3%)	0.328
*Genital area slapped*				
Past year	20% (12.7%, 30.2%)	19.5% (11%, 32.1%)	13.5% (4.7%, 33.1%)	0.503
Lifetime	27.9% (19.4%, 38.3%)	25.9% (16.1%, 39%)	23.2% (11%, 42.7%)	0.786
*Spanked lightly*				
Past year	36.6% (27%, 47.4%)	39.6% (27.6%, 53%)	23.7% (11%, 43.8%)	0.153
Lifetime	63.4% (52.4%, 73.2%)	67% (53.2%, 78.4%)	52.8% (33.5%, 71.2%)	0.219
*Spanked hard enough to leave a mark*				
Past year	18.1% (11.2%, 27.9%)	18.5% (10.5%, 30.4%)	8.2% (1.9%, 28.6%)	0.237
Lifetime	39.8% (30%, 50.5%)	40.2% (28.3%, 53.3%)	34.2% (18.6%, 54.2%)	0.598
*Choked*				
Past year	23% (15.1%, 33.4%)	25.7% (15.9%, 38.6%)	8.2% (1.9%, 28.6%)	0.075
Lifetime	41.3% (31.2%, 52.1%)	42.9% (30.5%, 56.2%)	32.7% (17.2%, 53.1%)	0.377
*Punched*				
Past year	4.4% (1.4%, 13.2%)	4.1% (1%, 15.9%)	0% (0%, 0%)	0.382
Lifetime	10% (5.1%, 18.8%)	8.7% (3.5%, 19.9%)	9.6% (2.8%, 28.1%)	0.889
*Called names*				
Past year	23.3% (15.3%, 33.8%)	26.2% (16.2%, 39.4%)	8% (2%, 26.7%)	0.054
Lifetime	42.6% (32.5%, 53.3%)	44.9% (32.4%, 58.1%)	32.4% (17.4%, 52.3%)	0.275
*Smothered during sexual activities*				
Past year	8.2% (3.7%, 16.9%)	8.1% (3.1%, 19.4%)	4.1% (0.5%, 25.3%)	0.512
Lifetime	16.3% (9.6%, 26.2%)	14.5% (7.3%, 26.5%)	11.5% (3.6%, 31.4%)	0.72
*Any of these with permission or consent*				
Past year	51.1% (40.6%, 61.5%)	55.1% (41.9%, 67.6%)	40.1% (23.1%, 59.9%)	0.203
Lifetime	72.3% (61.5%, 81.1%)	74.6% (60.8%, 84.8%)	65.6% (44.6%, 81.9%)	0.416

### TGNB+ Participants’ Reports of Receiving Rough Sex Behaviors, with Permission or Consent

Table 4 also shows the percentage of TGNB+ participants who reported that partner(s) had done these sexual behaviors to them. We found that 72.3% of TGNB+ participants (CI: 61.5%, 81.1%) reported that a sexual partner had done one or more of the sexual practices assessed to them (with permission or consent), ever in their lives, and that 51.1% (CI: 40.6%, 61.5%) reported having experienced one or more of these consensual behaviors in the prior year. The five most commonly reported lifetime behaviors that a partner had done to them, with permission or consent, were that they had: been spanked lightly (63.4%), been bitten (58.7%), had their hair pulled (56.8%), been called names (42.6%), and been spanked hard enough to leave a mark (39.8%).

### Rough Sex Engagement by Gender

As shown in Table 5, there were significant gender differences for past year and lifetime reports of enacting and receiving (with permission or consent) each of the assessed behaviors. As an example, having ever spanked a partner hard enough to leave a mark was reported by 37.4% of TGNB+ participants, 19.5% of men, and 8.5% of women (*p* < 0.001), with past year reports of hard spanking following a similar pattern. As a second example, 41.3% of TGNB+ participants, 15.8% of women, and 7.2% of men reported having ever been choked during sex, with past year reports following a similar pattern. Overall, women were significantly less likely than men or TGNB+ participants to enact one or more rough sex behaviors assessed on their partner(s) and women and TGNB+ participants were significantly more likely than men to be on the receiving end of one or more rough sex behaviors, with permission or consent. See Table 5 for details.

**Table 5:** Rough sex experiences reported by gender

	Women	Men	TGNB+	*p*-value
	*N* = 4562	*N* = 4346	*N* = 121	
*Participant did this to a partner*				
*Pulled their hair*				
Past year	14.9% (13.7%, 16.3%)	22.1% (20.6%, 23.6%)	34.9% (25.5%, 45.5%)	< 0.001
Lifetime	27.4% (25.9%, 29%)	39.6% (37.9%, 41.3%)	56.8% (46.1%, 66.9%)	< 0.001
*Bit them*				
Past year	17.1% (15.8%, 18.5%)	17.3% (16%, 18.8%)	38.9% (29.2%, 49.6%)	< 0.001
Lifetime	31.2% (29.6%, 32.8%)	33.1% (31.4%, 34.8%)	62.5% (51.6%, 72.2%)	< 0.001
*Slapped their face*				
Past year	2.5% (2%, 3.1%)	4.1% (3.4%, 4.9%)	17.8% (11%, 27.5%)	< 0.001
Lifetime	5.2% (4.5%, 6%)	8.9% (7.9%, 10%)	26.4% (18.1%, 36.9%)	< 0.001
*Slapped their genital area*				
Past year	5.1% (4.3%, 5.9%)	9.8% (8.8%, 11%)	16.5% (10%, 26.2%)	< 0.001
Lifetime	8.6% (7.7%, 9.6%)	18.3% (16.9%, 19.7%)	29% (20.4%, 39.5%)	< 0.001
*Spanked them lightly*				
Past year	18% (16.6%, 19.4%)	33.1% (31.4%, 34.9%)	36.4% (26.9%, 47.2%)	< 0.001
Lifetime	29.5% (27.9%, 31.1%)	53% (51.3%, 54.8%)	55.4% (44.6%, 65.7%)	< 0.001
*Spanked hard enough to leave a mark*				
Past year	4.3% (3.7%, 5.1%)	11% (9.9%, 12.2%)	23.3% (15.5%, 33.4%)	< 0.001
Lifetime	8.5% (7.6%, 9.5%)	19.5% (18.1%, 21%)	37.4% (27.9%, 48%)	< 0.001
*Choked them*				
Past year	6.2% (5.4%, 7.2%)	9.7% (8.7%, 10.9%)	23.8% (15.8%, 34.2%)	< 0.001
Lifetime	9.2% (8.3%, 10.3%)	15.8% (14.5%, 17.2%)	38.8% (29.1%, 49.6%)	< 0.001
*Punched them*				
Past year	1.0% (0.7%, 1.4%)	1.0% (0.6%, 1.4%)	10.0% (5%, 19.1%)	< 0.001
Lifetime	1.8% (1.4%, 2.3%)	1.9% (1.5%, 2.5%)	10.8% (5.6%, 19.8%)	< 0.001
*Called them names*				
Past year	3.3% (2.7%, 4.1%)	6.7% (5.8%, 7.6%)	20.2% (12.9%, 30.2%)	< 0.001
Lifetime	5.9% (5.1%, 6.8%)	13.1% (12%, 14.3%)	35.2% (25.8%, 45.9%)	< 0.001
*Smothered*				
Past year	1.4% (1%, 1.9%)	2.6% (2%, 3.3%)	10.9% (5.5%, 20.4%)	< 0.001
Lifetime	2.4% (1.9%, 3.1%)	4.4% (3.7%, 5.3%)	19.6% (12.4%, 29.6%)	< 0.001
*Did any of these to a partner*				
Past year	29.5% (27.9%, 31.1%)	39.2% (37.5%, 41%)	49% (38.6%, 59.5%)	< 0.001
Lifetime	47.8% (46.1%, 49.5%)	60.8% (59.1%, 62.5%)	71.1% (60.3%, 80%)	< 0.001
*Done to participant with consent*				
Hair was pulled				
Past year	20.8% (19.4%, 22.3%)	8.7% (7.7%, 9.8%)	28.9% (20.2%, 39.4%)	< 0.001
Lifetime	33.8% (32.2%, 35.5%)	15.8% (14.5%, 17.1%)	56.8% (46.1%, 66.9%)	< 0.001
*Bitten*				
Past year	15.3% (14%, 16.6%)	15% (13.7%, 16.4%)	29.8% (21.1%, 40.3%)	0.001
Lifetime	26.6% (25.1%, 28.1%)	29.8% (28.2%, 31.4%)	58.7% (47.9%, 68.7%)	< 0.001
*Face slapped*				
Past year	2.9% (2.4%, 3.6%)	2.7% (2.1%, 3.3%)	14.6% (8.4%, 24.1%)	< 0.001
Lifetime	5.9% (5.1%, 6.8%)	5.6% (4.9%, 6.5%)	23.2% (15.5%, 33.2%)	< 0.001
*Genital area slapped*				
Past year	8.7% (7.7%, 9.8%)	4.8% (4%, 5.6%)	20.0% (12.7%, 30.2%)	< 0.001
Lifetime	14% (12.9%, 15.3%)	9.5% (8.5%, 10.6%)	27.9% (19.4%, 38.3%)	< 0.001
*Spanked lightly*				
Past year	28% (26.4%, 29.6%)	14.6% (13.4%, 15.9%)	36.6% (27%, 47.4%)	< 0.001
Lifetime	43.5% (41.8%, 45.3%)	25.8% (24.3%, 27.4%)	63.4% (52.4%, 73.2%)	< 0.001
*Spanked hard enough to leave* a *mark*				
Past year	9.8% (8.8%, 11%)	4.4% (3.7%, 5.3%)	18.1% (11.2%, 27.9%)	< 0.001
Lifetime	16.3% (15.1%, 17.7%)	9.0% (8.1%, 10.1%)	39.8% (30%, 50.5%)	< 0.001
*Choked*				
Past year	10.9% (9.8%, 12.1%)	4.3% (3.6%, 5.2%)	23% (15.1%, 33.4%)	< 0.001
Lifetime	15.8% (14.5%, 17.1%)	7.2% (6.3%, 8.2%)	41.3% (31.2%, 52.1%)	< 0.001
*Punched*				
Past year	0.7% (0.5%, 1.1%)	1.1% (0.7%, 1.5%)	4.4% (1.4%, 13.2%)	0.003
Lifetime	1.6% (1.3%, 2.2%)	2.2% (1.7%, 2.8%)	10.0% (5.1%, 18.8%)	< 0.001
*Called names*				
Past year	5.7% (5%, 6.6%)	3.5% (2.9%, 4.2%)	23.3% (15.3%, 33.8%)	< 0.001
Lifetime	11.5% (10.5%, 12.7%)	7.4% (6.5%, 8.4%)	42.6% (32.5%, 53.3%)	< 0.001
Smothered				
Past year	2.3% (1.8%, 2.9%)	1.2% (0.9%, 1.7%)	8.2% (3.7%, 16.9%)	< 0.001
Lifetime	3.9% (3.2%, 4.6%)	2.5% (2%, 3.1%)	16.3% (9.6%, 26.2%)	< 0.001
*Any of these with permission or consent*				
Past year	34.6% (32.9%, 36.3%)	26.5% (25%, 28.1%)	51.1% (40.6%, 61.5%)	< 0.001
Lifetime	53.8% (52.1%, 55.5%)	45.8% (44%, 47.5%)	72.3% (61.5%, 81.1%)	< 0.001

### Women’s Reports of Nonconsensual Rough Sex Experiences

As shown in Table 6, 19.6% of women (95% CI: 18.3%, 21.0%) reported that someone had ever enacted one or more of the rough sex behaviors we assessed, without their permission or consent. Also, 8.5% of women (95% CI: 7.5%, 9.5%) reported that this had occurred in the prior year. The five most commonly reported nonconsensual lifetime experiences were having: been spanked lightly (11.4%), had their hair pulled (8.4%), been called names like bitch, slut, whore, or fag (6.8%), been bitten (6.7%), or been spanked hard enough to leave a mark (6.0%).

**Table 6:** Women's experiences of non-consensual rough sex by sexual orientation identity and age

	Overall	Sexual Orientation				Age							
		Heterosexual	Bisexual/Pansexual	Lesbian or Gay	*p*-value	18–24	25–29	30–39	40–49	50–59	60–69	70 +	*p*-value
	*N* = 4562	*N* = 4057	*N* = 271	*N* = 90		*N* = 372	*N* = 494	*N* = 711	*N* = 760	*N* = 768	*N* = 804	*N* = 653	
*Hair pulled*													
Past year	2.7% (2.2%, 3.4%)	2.5% (1.9%, 3.2%)	6.2% (3.6%, 10.7%)	2.9% (0.9%, 9.2%)	0.004	5% (2.6%, 9.5%)	2.7% (1.3%, 5.5%)	5% (3.3%, 7.5%)	4.5% (2.9%, 6.9%)	1.6% (0.9%, 2.9%)	0.6% (0.2%, 1.2%)	0.8% (0.3%, 1.8%)	< 0.001
Lifetime	8.4% (7.5%, 9.4%)	7.6% (6.7%, 8.7%)	18.4% (14.1%, 23.6%)	6.6% (3%, 13.7%)	< 0.001	9.5% (6.1%, 14.5%)	10.3% (7.1%, 14.7%)	15.2% (12.4%, 18.5%)	12.3% (9.8%, 15.4%)	7.6% (5.8%, 9.9%)	2.8% (1.9%, 4.3%)	1.9% (1.1%, 3.2%)	< 0.001
*Bit*													
Past year	2.3% (1.8%, 2.9%)	1.9% (1.5%, 2.6%)	7.9% (4.8%, 12.8%)	0.5% (0.1%, 3.6%)	< 0.001	4.7% (2.4%, 8.9%)	2.7% (1.3%, 5.3%)	3.4% (2.2%, 5.4%)	3.3% (2%, 5.3%)	1.1% (0.5%, 2.6%)	1.1% (0.6%, 2.2%)	0.8% (0.3%, 1.9%)	0.001
Lifetime	6.7% (5.9%, 7.6%)	5.9% (5.1%, 6.9%)	16.4% (12.2%, 21.7%)	5.1% (2.3%, 11.1%)	< 0.001	7% (4.1%, 11.7%)	9.3% (6.3%, 13.6%)	9.4% (7.2%, 12.2%)	9.8% (7.5%, 12.5%)	6.2% (4.5%, 8.4%)	3.5% (2.4%, 5.2%)	2.1% (1.3%, 3.4%)	< 0.001
*Slapped face*													
Past year	1.3% (1%, 1.8%)	1.1% (0.8%, 1.6%)	4.5% (2.2%, 8.9%)	1.3% (0.2%, 9%)	< 0.001	2.3% (0.9%, 5.7%)	1.4% (0.5%, 3.9%)	2.8% (1.6%, 4.7%)	1.4% (0.7%, 2.9%)	0.2% (0.1%, 0.9%)	0.7% (0.3%, 1.5%)	0.9% (0.4%, 1.9%)	0.005
Lifetime	4.2% (3.5%, 4.9%)	3.6% (3%, 4.4%)	11.4% (7.9%, 16.1%)	4.8% (1.9%, 11.2%)	< 0.001	8.0% (4.8%, 13%)	6.7% (4.1%, 10.7%)	7.5% (5.6%, 10%)	3.6% (2.4%, 5.3%)	1.7% (1%, 2.9%)	2.9% (1.8%, 4.4%)	1.7% (1%, 3%)	< 0.001
*Slapped genitals*													
Past year	1.6% (1.2%, 2.1%)	1.4% (1%, 1.9%)	4.1% (2.1%, 8%)	0% (0%, 0%)	0.005	3.2% (1.4%, 7.1%)	1.7% (0.7%, 4.3%)	2.3% (1.3%, 4%)	2.3% (1.3%, 4%)	1.0% (0.4%, 2.4%)	0.6% (0.2%, 1.7%)	0.6% (0.2%, 1.5%)	0.026
Lifetime	4.0% (3.4%, 4.8%)	3.6% (3%, 4.4%)	9.3% (6.3%, 13.6%)	4.1% (1.4%, 11.5%)	< 0.001	4.5% (2.3%, 8.4%)	4.3% (2.5%, 7.4%)	6.4% (4.6%, 8.9%)	5.8% (4.1%, 8.2%)	3.9% (2.6%, 5.6%)	1.5% (0.8%, 2.7%)	2.2% (1.3%, 3.6%)	< 0.001
*Spanked lightly*													
Past year	4.8% (4%, 5.7%)	4.9% (4.1%, 5.8%)	6.1% (3.6%, 10.1%)	1.0% (0.2%, 5.9%)	0.169	5.6% (3%, 10.3%)	6.6% (4%, 10.7%)	6.9% (5%, 9.6%)	8.4% (6.2%, 11.4%)	3.1% (1.8%, 5.1%)	1.9% (1.2%, 3.2%)	1.8% (1%, 3.2%)	< 0.001
Lifetime	11.4% (10.3%, 12.5%)	11.0% (9.8%, 12.2%)	17.8% (13.6%, 22.9%)	9.0% (4.9%, 16%)	0.002	9.1% (5.7%, 14.3%)	12.6% (8.9%, 17.4%)	15.4% (12.5%, 18.8%)	15.1% (12.2%, 18.4%)	13.5% (10.9%, 16.6%)	7.6% (5.9%, 9.7%)	4.9% (3.5%, 6.9%)	< 0.001
*Spanked hard*													
Past year	1.9% (1.5%, 2.5%)	1.8% (1.3%, 2.4%)	4.7% (2.6%, 8.4%)	2.4% (0.6%, 9.2%)	0.008	4.7% (2.5%, 8.9%)	2.9% (1.4%, 6%)	2.9% (1.8%, 4.7%)	2.8% (1.6%, 4.7%)	0.7% (0.2%, 1.8%)	0.5% (0.2%, 1.2%)	0.7% (0.3%, 2%)	< 0.001
Lifetime	6.0% (5.2%, 6.9%)	5.3% (4.5%, 6.2%)	14.5% (10.8%, 19.2%)	5.7% (2.6%, 12.2%)	< 0.001	7.5% (4.5%, 12.2%)	9.9% (6.7%, 14.4%)	9.9% (7.7%, 12.6%)	7.0% (5.2%, 9.4%)	5.2% (3.7%, 7.3%)	2.4% (1.5%, 3.8%)	1.6% (0.9%, 3%)	< 0.001
*Choked*													
Past year	1.9% (1.4%, 2.5%)	1.7% (1.2%, 2.3%)	5.2% (2.9%, 9.3%)	0% (0%, 0%)	0.001	5% (2.6%, 9.4%)	3.1% (1.5%, 6.3%)	2.8% (1.7%, 4.7%)	3.1% (1.9%, 5.1%)	0.2% (0%, 1.2%)	0.4% (0.1%, 1.1%)	0.4% (0.1%, 1.4%)	< 0.001
Lifetime	5.0% (4.3%, 5.8%)	4.3% (3.5%, 5.1%)	13.5% (9.8%, 18.3%)	7.5% (3.3%, 16.1%)	< 0.001	9.5% (6%, 14.6%)	9.4% (6.4%, 13.7%)	8% (6%, 10.5%)	7.4% (5.5%, 9.9%)	2% (1.1%, 3.4%)	1.4% (0.7%, 2.6%)	0.9% (0.4%, 2%)	< 0.001
*Punched*													
Past year	0.8% (0.5%, 1.2%)	0.7% (0.4%, 1%)	3.2% (1.4%, 7.3%)	0% (0%, 0%)	0.001	2.7% (1.1%, 6.5%)	0.2% (0%, 1.3%)	1.5% (0.7%, 3.2%)	1.3% (0.6%, 2.6%)	0.1% (0%, 0.6%)	0.2% (0%, 0.8%)	0.5% (0.2%, 1.5%)	< 0.001
Lifetime	2.3% (1.9%, 3%)	2.0% (1.5%, 2.6%)	5.3% (3%, 9.1%)	4.4% (1.7%, 10.8%)	0.001	3.9% (1.8%, 8.2%)	3.2% (1.6%, 6.6%)	3.5% (2.2%, 5.4%)	3.5% (2.2%, 5.4%)	0.8% (0.4%, 1.9%)	1.3% (0.7%, 2.4%)	1.3% (0.7%, 2.6%)	0.005
*Called names*													
Past year	1.9% (1.5%, 2.5%)	1.8% (1.3%, 2.4%)	5.0% (2.7%, 9.1%)	2.4% (0.6%, 9.1%)	0.005	3.6% (1.7%, 7.5%)	2.7% (1.3%, 5.7%)	2.9% (1.7%, 5%)	2.8% (1.8%, 4.5%)	0.8% (0.4%, 1.9%)	1.1% (0.5%, 2.1%)	0.7% (0.3%, 1.6%)	0.004
Lifetime	6.8% (6%, 7.7%)	6.1% (5.2%, 7%)	15.9% (11.9%, 20.9%)	10.5% (5.6%, 18.8%)	< 0.001	8.1% (5%, 12.9%)	10.4% (7.3%, 14.7%)	9.5% (7.3%, 12.2%)	7.6% (5.7%, 10%)	5.9% (4.2%, 8%)	4.0% (2.8%, 5.7%)	3.8% (2.6%, 5.5%)	< 0.001
*Smothered*													
Past year	0.8% (0.6%, 1.2%)	0.7% (0.4%, 1%)	2.8% (1.2%, 6.3%)	2.4% (0.6%, 9.2%)	0.001	2.1% (0.8%, 5.5%)	0.5% (0.1%, 2.1%)	1.9% (1%, 3.5%)	0.9% (0.4%, 2%)	0% (0%, 0.1%)	0.4% (0.1%, 1.1%)	0.6% (0.2%, 1.6%)	0.002
Lifetime	2.3% (1.8%, 2.9%)	2.0% (1.5%, 2.6%)	5.5% (3.3%, 9%)	2.9% (0.9%, 9.3%)	0.001	4.1% (2%, 8.1%)	3.5% (1.8%, 6.8%)	4.2% (2.9%, 6.2%)	3.1% (2%, 4.9%)	0.7% (0.3%, 1.7%)	0.8% (0.4%, 1.7%)	1.0% (0.5%, 2%)	< 0.001
*Any of these without permission or consent*													
Past year	8.5% (7.5%, 9.5%)	8.2% (7.2%, 9.3%)	15% (10.9%, 20.4%)	5.0% (2%, 11.6%)	0.001	10.3% (6.7%, 15.5%)	11.5% (8%, 16.2%)	11.6% (9.1%, 14.7%)	12.7% (10%, 16%)	5.8% (4%, 8.2%)	4.7% (3.4%, 6.4%)	4.4% (3.1%, 6.3%)	< 0.001
Lifetime	19.6% (18.3%, 21.0%)	18.3% (16.9%, 19.8%)	33.4% (27.8%, 39.5%)	20.7% (13.6%, 30.2%)	< 0.001	17.6% (12.8%, 23.7%)	24.7% (19.7%, 30.4%)	25.2% (21.7%, 29%)	24.3% (20.8%, 28.1%)	21.1% (18%, 24.7%)	13.2% (11%, 15.8%)	11% (8.8%, 13.6%)	< 0.001

In terms of sexual orientation identity, there were significant differences for each of the ten nonconsensual experiences assessed. As one example, lifetime experience of nonconsensual choking was reported by 13.5% of bisexual/pansexual women, 7.5% of lesbian women, and 4.3% of heterosexual women (*p* < 0.001). As a second example, 11.4% of bisexual/pansexual women, 4.8% of lesbian women, and 3.6% of heterosexual women reported having been slapped on their face without consent. As a third example, nonconsensual punching was reported by 5.3% of bisexual/pansexual women, 4.4% of lesbian women, and 2.0% of heterosexual women (*p* < 0.001).

In terms of age, each of the ten nonconsensual behaviors was significantly more likely to be reported by younger women as compared with older women. For example, having ever been choked without permission or consent was reported by 7.4% to 9.5% of women between the ages of 18 and 49 but by 0.9% to 2.0% of women ages 50 and older (*p* < 0.001). We also found that while having been spanked hard enough to leave a mark was relatively rare among women in their 60 s (2.4%) and ages 70 + (1.6%), it was significantly more common among younger women, reported by 7.0% to 9.9% of women ages 18 to 49 and 5.2% of women in their 50s.

### Men’s Reports of Nonconsensual Rough Sex Experiences

As shown in Table 7, 15.9% of men (95% CI: 14.6%, 17.2%) reported that someone had ever done one or more of the rough sex behaviors assessed without their permission or consent. Also, 7.4% of men (95% CI: 6.5%, 8.5%) reported that this had occurred in the prior year. The five most commonly reported nonconsensual lifetime experiences were having: been bitten (9.2%), been spanked lightly (7.9%), had their hair pulled (4.9%), had their genitals slapped (4.1%), and had their face slapped (3.5%).

**Table 7:** Men's experiences of non-consensual rough sex by sexual orientation identity and age

	Overall	Sexual Orientation				Age							
		Heterosexual	Bisexual/Pansexual	Lesbian or Gay	*p*-value	18–24	25–29	30–39	40–49	50–59	60–69	70 +	*p*-value
	*N* = 4346	*N* = 3933	*N* = 100	*N* = 187		*N* = 425	*N* = 443	*N* = 689	*N* = 785	*N* = 743	*N* = 681	*N* = 580	
*Hair pulled*													
Past year	2.5% (2%, 3.2%)	2.3% (1.7%, 3%)	5.6% (2.3%, 12.9%)	3% (1.3%, 6.4%)	0.093	2.4% (0.9%, 6.5%)	4.4% (2.3%, 8.2%)	3.5% (2.2%, 5.5%)	3.5% (2.2%, 5.6%)	2.5% (1.3%, 4.6%)	0.8% (0.3%, 1.9%)	0.6% (0.2%, 1.9%)	0.01
Lifetime	4.9% (4.1%, 5.7%)	4.5% (3.7%, 5.4%)	10.3% (5.6%, 18.3%)	7% (4.3%, 11.1%)	0.007	5% (2.5%, 9.8%)	6% (3.5%, 10.1%)	7.4% (5.3%, 10.1%)	6.1% (4.3%, 8.6%)	4.3% (2.8%, 6.4%)	3.1% (2%, 4.6%)	2.2% (1.3%, 3.7%)	0.01
*Bit*													
Past year	3.8% (3.2%, 4.7%)	3.8% (3%, 4.6%)	4.6% (1.8%, 11.6%)	3.8% (2%, 7.3%)	0.898	3.7% (1.7%, 8.1%)	5.7% (3.2%, 9.9%)	6.4% (4.4%, 9.3%)	5.3% (3.6%, 7.7%)	2.8% (1.7%, 4.6%)	1.7% (0.9%, 3.1%)	1.4% (0.8%, 2.6%)	0.001
Lifetime	9.2% (8.2%, 10.2%)	8.9% (7.8%, 10%)	12.3% (7.1%, 20.4%)	13% (9.1%, 18.2%)	0.082	6.6% (3.6%, 11.7%)	9% (5.9%, 13.6%)	12.5% (9.7%, 15.8%)	10.4% (8.1%, 13.4%)	10.7% (8.5%, 13.5%)	6.9% (5.2%, 9.2%)	5.9% (4.3%, 7.9%)	0.009
*Slapped face*													
Past year	1.5% (1.1%, 2.1%)	1.4% (1%, 2%)	2.4% (0.7%, 7.8%)	3.4% (1.6%, 7%)	0.081	2.4% (0.9%, 6.6%)	4% (2.1%, 7.7%)	3.0% (1.8%, 5%)	1.5% (0.8%, 2.8%)	0.1% (0%, 0.5%)	0.3% (0.1%, 1.2%)	0.5% (0.2%, 1.4%)	< 0.001
Lifetime	3.5% (2.9%, 4.2%)	3.1% (2.5%, 3.9%)	10.1% (5.5%, 17.8%)	7.3% (4.5%, 11.5%)	< 0.001	4.7% (2.3%, 9.5%)	5.5% (3.2%, 9.3%)	6.4% (4.5%, 9%)	3.9% (2.5%, 6%)	2.1% (1.3%, 3.5%)	1.2% (0.6%, 2.3%)	1.6% (0.9%, 2.7%)	< 0.001
*Slapped genitals*													
Past year	2.2% (1.7%, 2.8%)	2.0% (1.6%, 2.7%)	1.8% (0.6%, 5.5%)	3.4% (1.6%, 7.2%)	0.351	2.5% (1%, 6.2%)	3.6% (1.7%, 7.3%)	3.9% (2.5%, 6.1%)	2.1% (1.2%, 3.7%)	1.7% (0.9%, 3.2%)	0.8% (0.3%, 1.8%)	1% (0.5%, 2.1%)	0.012
Lifetime	4.1% (3.4%, 4.8%)	3.8% (3.1%, 4.6%)	7.4% (3.5%, 15%)	7.7% (5%, 11.8%)	0.006	3.7% (1.7%, 7.9%)	4.3% (2.3%, 8%)	6.3% (4.4%, 8.9%)	4.4% (2.9%, 6.6%)	4.0% (2.7%, 6%)	2.6% (1.7%, 4.1%)	2.8% (1.8%, 4.3%)	0.13
*Spanked lightly*													
Past year	3.8% (3.2%, 4.6%)	3.5% (2.8%, 4.3%)	6.9% (3%, 15%)	7.1% (4.3%, 11.4%)	0.015	2.3% (0.8%, 6.2%)	2.5% (1.2%, 4.8%)	6.1% (4.3%, 8.5%)	6.4% (4.5%, 9%)	3.8% (2.4%, 6%)	2.5% (1.5%, 4%)	1.2% (0.6%, 2.3%)	< 0.001
Lifetime	7.9% (7%, 8.9%)	7.2% (6.3%, 8.3%)	15.7% (9.5%, 24.7%)	17.3% (12.8%, 23%)	< 0.001	5% (2.5%, 9.7%)	4.2% (2.5%, 7.2%)	9.9% (7.5%, 12.8%)	10.9% (8.4%, 13.9%)	9.3% (7.1%, 12%)	7% (5.3%, 9.3%)	5.5% (3.9%, 7.6%)	0.001
*Spanked hard*													
Past year	1.3% (1%, 1.8%)	1.1% (0.8%, 1.5%)	2.2% (0.6%, 7.7%)	3.8% (1.9%, 7.4%)	0.003	0% (0%, 0%)	2.2% (0.9%, 5%)	2.9% (1.8%, 4.7%)	1.9% (1%, 3.4%)	0.9% (0.4%, 1.9%)	0.3% (0.1%, 1.2%)	0.7% (0.3%, 1.8%)	< 0.001
Lifetime	3.4% (2.8%, 4.2%)	2.9% (2.3%, 3.6%)	11% (5.8%, 19.8%)	8.2% (5.2%, 12.7%)	< 0.001	2.7% (1.1%, 6.8%)	3.9% (2.1%, 7.2%)	6.6% (4.7%, 9.3%)	4.5% (3.1%, 6.6%)	2.5% (1.5%, 3.9%)	1.7% (0.8%, 3.4%)	1.6% (0.9%, 2.9%)	< 0.001
*Choked*													
Past year	1.1% (0.8%, 1.5%)	0.9% (0.6%, 1.4%)	4.2% (1.4%, 11.9%)	2.4% (1%, 5.5%)	0.004	1.6% (0.5%, 4.9%)	1.5% (0.6%, 3.9%)	2.5% (1.4%, 4.4%)	1.1% (0.6%, 2.3%)	0.4% (0.1%, 1.7%)	0.3% (0.1%, 1.2%)	0.4% (0.1%, 1.4%)	0.009
Lifetime	2.4% (1.9%, 3%)	2.2% (1.7%, 2.8%)	6.3% (2.8%, 13.4%)	5.1% (3%, 8.6%)	0.001	3.2% (1.4%, 7.2%)	2% (0.9%, 4.5%)	5.6% (3.9%, 8.1%)	2.7% (1.6%, 4.5%)	1% (0.5%, 2.1%)	1.3% (0.6%, 2.5%)	1% (0.5%, 2.1%)	< 0.001
*Punched*													
Past year	0.8% (0.5%, 1.2%)	0.8% (0.5%, 1.2%)	0.6% (0.1%, 4.4%)	2.2% (1%, 4.9%)	0.046	1.2% (0.3%, 5%)	0.5% (0.1%, 2.4%)	2.1% (1%, 4%)	0.8% (0.3%, 1.7%)	0.2% (0%, 1.2%)	0.3% (0.1%, 1.2%)	0.7% (0.2%, 1.7%)	0.027
Lifetime	1.8% (1.4%, 2.4%)	1.7% (1.3%, 2.4%)	1.3% (0.3%, 4.9%)	3.3% (1.7%, 6.2%)	0.165	2.5% (0.9%, 6.6%)	1.2% (0.3%, 4.1%)	4.7% (3%, 7.1%)	1.6% (0.8%, 3.2%)	0.7% (0.3%, 1.5%)	1.2% (0.6%, 2.4%)	0.8% (0.3%, 1.8%)	< 0.001
*Called names*													
Past year	1.1% (0.8%, 1.6%)	0.9% (0.6%, 1.3%)	4.1% (1.4%, 11.5%)	4.7% (2.5%, 8.4%)	< 0.001	0.8% (0.1%, 5.3%)	2.2% (1%, 4.7%)	2.5% (1.4%, 4.3%)	1.1% (0.5%, 2.2%)	0.5% (0.2%, 1.4%)	0.7% (0.3%, 1.7%)	0.5% (0.2%, 1.2%)	0.032
Lifetime	3.4% (2.9%, 4.1%)	2.8% (2.2%, 3.5%)	11.8% (6.6%, 20.3%)	11.9% (8.3%, 16.8%)	< 0.001	3.3% (1.4%, 7.5%)	3.9% (2.2%, 6.9%)	6% (4.1%, 8.6%)	3.1% (2%, 5%)	2.7% (1.8%, 4.1%)	3.1% (2%, 4.8%)	1.9% (1.2%, 3%)	0.034
*Smothered*													
Past year	0.7% (0.4%, 1.1%)	0.7% (0.4%, 1.1%)	0.8% (0.1%, 4.2%)	1.1% (0.3%, 3.7%)	0.706	0% (0%, 0%)	0.1% (0%, 1.1%)	2.4% (1.2%, 4.5%)	0.8% (0.3%, 2%)	0.2% (0%, 1%)	0.5% (0.2%, 1.4%)	0.3% (0.1%, 0.9%)	< 0.001
Lifetime	1.8% (1.3%, 2.4%)	1.7% (1.2%, 2.3%)	2% (0.7%, 5.7%)	4.4% (2.3%, 8.5%)	0.013	3.2% (1.3%, 7.7%)	1.5% (0.5%, 4.3%)	4.1% (2.6%, 6.5%)	2% (1.1%, 3.6%)	0.2% (0.1%, 1%)	1.1% (0.5%, 2.3%)	0.8% (0.4%, 1.7%)	< 0.001
*Any of these without permission* or *consent*													
Past year	7.4% (6.5%, 8.5%)	7% (6%, 8.1%)	10.8% (5.7%, 19.7%)	10.5% (7.2%, 15.2%)	0.081	5.5% (2.9%, 10.2%)	7.6% (4.8%, 12%)	10.7% (8.1%, 13.8%)	10.3% (7.9%, 13.3%)	7.1% (5.1%, 9.8%)	4.5% (3.1%, 6.3%)	4.7% (3.4%, 6.6%)	0.001
Lifetime	15.9% (14.6%, 17.2%)	15.1% (13.8%, 16.5%)	27.6% (19.3%, 37.8%)	24.3% (19.2%, 30.2%)	< 0.001	10.3% (6.4%, 16.2%)	12.1% (8.5%, 17%)	19.3% (16%, 23.1%)	18.2% (15.2%, 21.7%)	18.3% (15.4%, 21.7%)	14.5% (11.9%, 17.4%)	13.9% (11.5%, 16.8%)	0.006

In terms of sexual orientation identity, there were statistically significant differences for some of the nonconsensual experiences assessed (both lifetime and past year). As one example, lifetime experience of being choked without consent was reported by 6.3% of bisexual/pansexual men, 5.1% of gay men, and 2.2% of heterosexual men (*p* < 0.001). As a second example, 7.7% of gay men, 7.4% of bisexual/pansexual men, and 3.8% of heterosexual men reported having been slapped on their genitals without consent (*p* < 0.01). As a third example, 11.8% of bisexual/pansexual men, 11.9% of gay men, and 2.8% of heterosexual men reported having been called names without consent or permission (*p* < 0.001).

In terms of age, each of the ten nonconsensual behaviors was significantly more likely to be reported by younger men as compared with older men. For example, having ever been slapped on the face without permission or consent was reported by 3.9% to 6.4% of men between the ages of 18 and 49 but by 1.2% to 2.1% of men ages 50 and older (*p* < 0.001).

### TGNB+ Participants’ Nonconsensual Rough Sex Experiences

As shown in Table 8, 35.3% (95% CI: 26.0%, 45.9%) of TGNB+ participants reported that someone had ever done at least one of the rough sex behaviors to them, but without their permission or consent and 15.5% (95% CI: 9.3%, 24.9%) reported that this had occurred in the past year. The five most commonly reported nonconsensual behaviors assessed were having: been bitten (24.8%), been called names (25.1%), had their hair pulled (25.0%), been spanked lightly (22.4%), and had their face slapped (22.1%). There were no significant differences for participants’ reports of past year or lifetime nonconsensual experiences with these behaviors, in relation to their birth assigned sex.

**Table 8:** Non-consensual rough sex behaviors reported by transgender, gender nonbinary, and other gender diverse (TGNB+) participants.

	Overall TGNB+	Gender/Sex Assigned at Birth		
		TGNB+/AFAB	TGNB+/AMAB	*p*-value
	*N* = 121	*N* = 83	*N* = 31	
*Done to participant without permission or consent*				
*Pulled their hair*				
Past year	6.6% (2.7%, 15%)	7.4% (2.7%, 18.3%)	0% (0%, 0%)	0.186
Lifetime	25.0% (17%, 35.2%)	21.5% (12.7%, 33.9%)	24.6% (11.8%, 44.3%)	0.74
*Bit them*				
Past year	10.1% (5%, 19.1%)	9.2% (3.8%, 20.7%)	8.2% (1.9%, 28.6%)	0.882
Lifetime	24.8% (16.8%, 35%)	25.2% (15.6%, 38%)	21.2% (9.6%, 40.7%)	0.686
*Slapped their face*				
Past year	8.4% (3.9%, 17.1%)	6.8% (2.4%, 17.8%)	8.2% (1.9%, 28.6%)	0.829
Lifetime	22.1% (14.5%, 32.2%)	20.2% (11.7%, 32.6%)	17.3% (7.2%, 36.1%)	0.743
*Slapped their genital area*				
Past year	7.3% (3.2%, 15.9%)	6.8% (2.4%, 17.8%)	4.1% (0.5%, 25.3%)	0.633
Lifetime	17.7% (11%, 27.3%)	18.7% (10.6%, 31%)	11.2% (3.6%, 29.6%)	0.368
*Spanked them lightly*				
Past year	8.6% (4.1%, 17.2%)	8.1% (3.1%, 19.2%)	5.7% (1.2%, 23.6%)	0.689
Lifetime	22.4% (14.9%, 32.1%)	24.4% (15.1%, 36.9%)	14.3% (5.4%, 32.7%)	0.271
*Spanked hard enough to leave a mark*				
Past year	5.5% (2%, 14.2%)	5.7% (1.8%, 17.2%)	0% (0%, 0%)	0.266
Lifetime	20% (12.8%, 29.9%)	16.6% (9%, 28.6%)	18.2% (7.7%, 37.4%)	0.852
*Choked them*				
Past year	9.1% (4.4%, 17.8%)	9.4% (4%, 20.4%)	4.1% (0.5%, 25.7%)	0.423
Lifetime	20.2% (12.9%, 30.2%)	19.8% (11.4%, 32.1%)	11% (3.4%, 30.4%)	0.316
*Punched them*				
Past year	4.9% (1.8%, 12.6%)	3.1% (0.8%, 11.9%)	4.2% (0.5%, 26%)	0.802
Lifetime	17.3% (10.4%, 27.2%)	14.6% (7.4%, 26.7%)	13.2% (4.6%, 32.5%)	0.866
*Called them names*				
Past year	9.8% (4.9%, 18.4%)	5.7% (1.7%, 17%)	16.2% (6.3%, 35.6%)	0.131
Lifetime	25.1% (17.1%, 35.2%)	24.4% (15.2%, 36.8%)	17.4% (7.2%, 36.5%)	0.459
*Smothered them during sexual activities*				
Past year	8.0% (3.6%, 17%)	7.8% (2.9%, 19.7%)	4.1% (0.5%, 25.3%)	0.54
Lifetime	14.7% (8.5%, 24.3%)	15.5% (8%, 27.8%)	8.2% (1.9%, 28.6%)	0.377
*Someone did at least one of these to the participant, without permission or consent*				
Past year	15.5% (9.3%, 24.9%)	14.2% (7.2%, 26.1%)	15.9% (6.2%, 35.1%)	0.837
Lifetime	35.3% (26.0%, 45.9%)	36.1% (24.8%, 49.2%)	26.2% (13%, 45.7%)	0.354

AFAB refers to those assigned female at birth and AMAB refers to those assigned male at birth

### Nonconsensual Rough Sex by Gender

With the exception of having had one’s hair pulled in the prior year, we observed significant differences by gender for each of the nonconsensual rough sex behaviors assessed (Table 9). We found that 35.3% of TGNB+ participants, 19.6% of women and 15.9% of men reported having ever experienced one or more of the rough sex behaviors assessed (*p* < 0.001), without permission or consent, as had 15.5% of TGNB+ participants, 8.5% of men, and 7.4% of women in the prior year (*p* < 0.02). TGNB+ participants were significantly more likely to report having ever experienced each nonconsensual behavior assessed. As one example, 22.1% of TGNB+ participants, 4.2% of women, and 3.5% of men reported having ever been slapped on their face, without permission or consent (*p* < 0.001). As a second example, 20.2% of TGNB+ participants, 5.0% of women, and 2.4% of men reported having ever been choked during sexual activities without consent (*p* < 0.001). Third, 17.3% of TGNB+ participants, 2.3% of women, and 1.8% of men reported that they had been punched during sexual activities, without permission or consent. See Table 9 for additional details.

**Table 9:** Non-consensual rough sex experiences by gender

	Women	Men	TGNB+	*p*-value
	*N* = 4562	*N* = 4346	*N* = 121	
*Pulled their hair*				
Past year	2.7% (2.2%, 3.4%)	2.5% (2%, 3.2%)	6.6% (2.7%, 15%)	0.102
Lifetime	8.4% (7.5%, 9.4%)	4.9% (4.1%, 5.7%)	25% (17%, 35.2%)	< 0.001
*Bit them*				
Past year	2.3% (1.8%, 2.9%)	3.8% (3.2%, 4.7%)	10.1% (5%, 19.1%)	< 0.001
Lifetime	6.7% (5.9%, 7.6%)	9.2% (8.2%, 10.2%)	24.8% (16.8%, 35%)	< 0.001
*Slapped their face*				
Past year	1.3% (1%, 1.8%)	1.5% (1.1%, 2.1%)	8.4% (3.9%, 17.1%)	< 0.001
Lifetime	4.2% (3.5%, 4.9%)	3.5% (2.9%, 4.2%)	22.1% (14.5%, 32.2%)	< 0.001
*Slapped their genital area*				
Past year	1.6% (1.2%, 2.1%)	2.2% (1.7%, 2.8%)	7.3% (3.2%, 15.9%)	0.001
Lifetime	4% (3.4%, 4.8%)	4.1% (3.4%, 4.8%)	17.7% (11%, 27.3%)	< 0.001
*Spanked them lightly*				
Past year	4.8% (4%, 5.7%)	3.8% (3.2%, 4.6%)	8.6% (4.1%, 17.2%)	0.033
Lifetime	11.4% (10.3%, 12.5%)	7.9% (7%, 8.9%)	22.4% (14.9%, 32.1%)	< 0.001
*Spanked them hard enough to leave a mark*				
Past year	1.9% (1.5%, 2.5%)	1.3% (1%, 1.8%)	5.5% (2%, 14.2%)	0.007
Lifetime	6% (5.2%, 6.9%)	3.4% (2.8%, 4.2%)	20% (12.8%, 29.9%)	< 0.001
*Choked them*				
Past year	1.9% (1.4%, 2.5%)	1.1% (0.8%, 1.5%)	9.1% (4.4%, 17.8%)	< 0.001
Lifetime	5.0% (4.3%, 5.8%)	2.4% (1.9%, 3%)	20.2% (12.9%, 30.2%)	< 0.001
*Punched them*				
Past year	0.8% (0.5%, 1.2%)	0.8% (0.5%, 1.2%)	4.9% (1.8%, 12.6%)	0.001
Lifetime	2.3% (1.9%, 3%)	1.8% (1.4%, 2.4%)	17.3% (10.4%, 27.2%)	< 0.001
*Called them names*				
Past year	1.9% (1.5%, 2.5%)	1.1% (0.8%, 1.6%)	9.8% (4.9%, 18.4%)	< 0.001
Lifetime	6.8% (6%, 7.7%)	3.4% (2.9%, 4.1%)	25.1% (17.1%, 35.2%)	< 0.001
*Smothered during sexual activities*				
Past year	0.8% (0.6%, 1.2%)	0.7% (0.4%, 1.1%)	8.0% (3.6%, 17%)	< 0.001
Lifetime	2.3% (1.8%, 2.9%)	1.8% (1.3%, 2.4%)	14.7% (8.5%, 24.3%)	< 0.001
*Any of these without permission or consent*				
Past year	8.5% (7.5%, 9.5%)	7.4% (6.5%, 8.5%)	15.5% (9.3%, 24.9%)	0.015
Lifetime	19.6% (18.3%, 21%)	15.9% (14.6%, 17.2%)	35.3% (26%, 45.9%)	< 0.001

## Discussion

The present study used data from the 2022 National Survey of Sexual Health and Behavior to provide population-level estimates on U.S. adults’ experiences with consensual and nonconsensual sexual practices commonly considered to be forms of “rough sex.” A first major finding is that most participants in this U.S. nationally representative survey of adults ages 18 to 94 had engaged in at least one of the sexual behaviors assessed, though the prevalence varied considerably by age group. Specifically, about 48% of women, 61% of men, and 71% of TGNB+ adults reported having ever done one or more of these to a partner, and about 54% of women, 46% of men, and 72% of TGNB+ adults reported that someone had done at least one of these sexual practices to them with permission or consent, differences that were statistically significant by gender.

These gendered patterns are generally consistent with prior research showing that more men and TGNB+ adults, as compared with women, tend to report having performed “rough sex” on their partner(s), whereas higher numbers of women and TGNB+ adults, as compared with men, report that a partner has done one or more of these behaviors to them, usually with consent (Döring et al., 2024; Herbenick et al., 2025; Holvoert et al., 2017; Sharman et al., 2025a, 2025b). In sex between women and men, women are more often on the receiving end of rough sex (e.g., are more often the ones who are choked, slapped, or spanked) and men more often choke, slap, or spank their partners (Herbenick et al., 2022b, 2022c; Holvoert et al., 2017). This may suggest that, whereas kink-identified people often engage with diverse sexual practices in ways that play with or subvert traditional gender roles or are more gender-flexible with participants’ roles in sex (Banerjee et al., 2018; Barker, 2013; Mollaioli et al., 2025), contemporary rough sex as portrayed and/or practiced in mainstream culture (the general population) may more closely adhere to and reproduce traditional gender roles related to sexual expression.

In regard to name calling (which varied both by gender and by sexual orientation identity), we had used the words bitch, slut, whore, and fag as an examples, which might be used more often against women and sexual minority men as compared with heterosexual men and thus may have influenced our findings. However, in the item-generation phase with faculty and students (and in cognitive interviews), no terms that might be more likely to be directed to heterosexual men emerged. Indeed, the extant literature notes that—whether in school bullying, sexual situations, or pornography—words that speak to women’s sexual reputation and that are common homophobic slurs are the words that tend to be employed (Espelage et al., 2018; Herbenick et al., 2020; Wright et al., 2015). Subsequent research is needed to understand how name-calling is used and experienced in both consensual and nonconsensual rough sex experiences.

A second major finding is that the sexual practices assessed in this study were significantly more often reported by younger adults as compared with older adults. This finding is consistent with prior research that has noted greater prevalence of rough sex among young people (Döring et al., 2024; Herbenick et al., 2017, 2020; Sharman et al., 2025a, 2025b; Vilhjálmsdóttir & Forberg, 2023). These age group differences may be related to references to and/or depictions of rough sex practices in mainstream media (e.g., growing up in the age of Fifty Shades of Grey), social media, internet memes, popular television shows, popular songs, fanfiction, online misinformation about certain sexual practices (e.g., those that describe “safe” ways to engage in choking), differences in the use of technologies for identifying partners, and broad access to online pornography through smartphones and tablet devices (Balle et al., 2024; Herbenick et al., 2022a, 2023b, 2023c; Keene et al., 2021; Wright et al., 2022a, 2023). Subsequent research is needed to understand emerging adults’ sexual experiences, especially given prior research showing that adults are having less partnered sex of various sorts (Herbenick et al., 2022e; Ueda et al., 2020), while at the same time, seem to be engaging in rough sex practices at higher rates than earlier generations.

Third, we found that gay and bisexual/pansexual participants were significantly more likely than heterosexual participants to report engaging in the consensual rough sex practices assessed. We also found that more TGNB+ participants reported that they had experienced consensual rough sex as compared with women or men. Our findings align with research examining the intersections of LGBTQ+ and kink identities, including that LGBTQ+ people more often report being kink-identified, engaging in kinky kinds of sex, and/or being open to exploring, playing with, and pushing back against expected gender roles and norms as a part of sex as compared with heterosexual people (Sprott, 2023; Sprott & Benoit Hadchock, 2018). Also, bisexual/pansexual women were significantly more likely than both heterosexual and lesbian women to be on the receiving end of consensual rough sex; this was especially pronounced for being choked and slapped on their genitals. Subsequent research might examine the extent to which these experiences are truly consensual, given that some research has found that people may describe choking as consensual even when it is not discussed or agreed to ahead of time and even when it is done as a form of sexual compliance or pleasing one’s partner (Herbenick et al., 2022c, 2025). However, given prior research showing higher rates of masturbation and vibrator use among bisexual women, for example (Bowman, 2014; Herbenick et al., 2009), it is also possible that the higher rates of consensual rough sex engagement among bisexual and pansexual identified women may reflect openness to sexual variety. Focused research on bisexual women and pansexual people is warranted and should consider how their identities, as well as the gender of their partner, may be related to their experiences with these sexual behaviors.

Fourth, our study extends the literature by providing population estimates on nonconsensual rough sex practices. We found that about 20% of women, 16% of men, and 35% of TGNB+ adults reported having experienced at least one of these behaviors without permission or consent. Nonconsensual rough sex was significantly more often reported by sexual minoritized women and men, such that 1 in 3 bisexual women and 1 in 4 gay and bisexual/pansexual men reported at least one nonconsensual rough sex experience. Taken together, study findings align with other research showing that sexual and gender minoritized individuals are significantly more likely than heterosexual and cisgender people to report having been sexually assaulted and that bisexual women and TGNB+ people are at especially high risk of assault (Eisenberg et al., 2021; Griner et al., 2020; Truman & Morgan, 2022). Subsequent research should examine nonconsensual rough sex experiences more closely, including the extent to which these nonconsensual rough sex experiences were consent violations within otherwise consensual sex versus from wholly nonconsensual encounters. Subsequent research should also interrogate if and to whom people disclose their nonconsensual rough sex experiences as well as how they navigate consent violations with established partners.

## Strengths and Limitations

Our study had several strengths. First, by collecting data from a U.S. nationally representative sample of adults across the lifespan, we were able to provide population estimates of diverse sexual practices, both for the prior year as well as lifetime engagement in each behavior and for young adulthood through advanced age, as well as demographic correlates of these behaviors. Both the sampling methods and the use of the statistical weights enhance the ability to generalize findings to the U.S. population of English and Spanish speaking adults. We also had a strong survey completion rate. Third, we asked participants whether they had done the behavior to another person (e.g., slapped a partner’s face during sexual activities) as well as whether a partner had done the behavior to them (e.g., if they had been slapped on the face during sexual activities). In doing so, we overcame a limitation of prior studies that did not distinguish between giving or receiving roles (Herbenick et al., 2017; Vilhjálmsdóttir & Forberg, 2023).

Another strength is that we surveyed adults across nine decades of life, supporting a life course perspective on examining engagement in these sexual behaviors. Finally, for sexual practices that had been done to participants, we assessed both consensual and nonconsensual experiences; subsequent research might examine people’s perceptions of the extent to which the rough sex behaviors they’ve done to their partners have been consensual, nonconsensual, or unclear in terms of consent.

Regarding our study’s limitations, although we assessed ten sexual practices and both giving and receiving roles, this is still a limited set of behaviors. We had to limit our assessment to be attentive to participant burden and due to survey cost. Subsequent research might examine a broader range of sexual practices and be attentive to changing norms and prevalent practices. Additionally, researchers might investigate the extent to which people enact rough sex as a suite of behaviors versus independently. Second, our study was limited to non-institutionalized U.S. adults who were able to complete surveys in English or Spanish languages. Research is needed that examines these sexual behaviors in countries around the world as well as by more culturally and linguistically diverse groups of U.S. adults. Third, given our relatively small sample of TGNB+ adults, we did not examine TGNB+ participants’ reports of rough sex by specific subgroups (e.g., transgender women, transgender men, nonbinary people, gender expansive people) or by sexual orientation; focused research on TGNB+ experiences with rough sex is warranted, especially given the high rates of both consensual and nonconsensual rough sex reported among TGNB+ participants.

Another limitation is that, although we assessed consent, we did not assess wantedness, feelings about, or sexual pleasure related to engaging in these behaviors, nor did we examine how these practices are organized into people’s sexual lives (e.g., whether these behaviors are enacted within established relationships or in casual relationship; whether they are part of individuals’ regular sexual repertoire or unusual acts). We also did not assess the timing of consent, given the difficulty of doing that over a lifetime of experiences, nor did we assess people’s reports of enacting rough sex on other people, without consent. Subsequent research should examine more detailed aspects of consent in relation to rough sex, including verbal and nonverbal consent cues as well as consent communication that occurs in advance of partnered sex as compared to within the sexual event itself.

Of note, we did not use the term “rough sex” with participants but instead simply asked about each behavior, as described above. However, subsequent research should examine how U.S. adults conceptualize rough sex, as current knowledge of this is largely based on college student samples (Burch & Salmon, 2022; Herbenick et al., 2021a; Svetina Valdivia et al., 2022). A fifth limitation is that we combined bisexual and pansexual participants together, which was an imperfect choice but one informed by the emerging literature on the similarities and differences between people who identify with these and other plurisexual terms (Galupo et al., 2018; Greaves et al., 2019). Subsequent research on rough sex might examine people’s experiences in ways that account for their intersecting identities. Finally, we did not include a severity index and so we do not know what proportion of people’s experiences with each of the 10 rough sex behaviors were done with light, moderate, or high intensities, nor do we know about potential impacts on their bodies or how they felt about these intensities. Subsequent research is needed in this area.

The 2022 NSSHB did collect data on adolescents’ experiences with rough sex but, due to space limitations as well as methodological issues (the adolescent sample was subject to different ways of asking about these sexual practices), those data will be presented separately. In addition, given calls from scholars to avoid atheoretical race-related comparisons, which have often been used in harmful ways (Hogarth, 2019), we did not examine these sexual practices by race/ethnicity in the present paper, though we plan to present focused (non-comparative) analyses related to race, ethnicity, and sexual behavior in the future.

## Implications

Our study has important implications for researchers, clinicians, and educators. First, given how prevalent and consequential rough sex behaviors have become and that they disproportionately affect marginalized groups (women, gender minorities, and sexual minorities), continued surveillance of these sexual practices through U.S. nationally representative survey research is critically needed. Such research should include behavioral surveillance but also address how people learn about these sexual behaviors, their potential health consequences, wantedness, pleasure, and the extent to which people engaging in these practices may be connected to kink and/or BDSM communities. This is important given kink and BDSM principles that speak to explicit consent, communication, discussion about boundaries and limits, education, risk awareness, and aftercare (Lee et al., 2015; Williams et al., 2014); thus, involvement in these communities may help to increase the likelihood that engagement in rough sex is practiced in a way that is safe and consensual.

Second, clinicians would benefit from receiving continuing education about these shifts in sexual behaviors; their relationship to mental health and physical health; and the importance of addressing diverse consensual sexual behaviors with their clients in non-shaming, nonjudgmental ways. After all, prior research has shown that stigma can be a barrier for people in terms of talking with their healthcare providers about sex-related injuries (Waldura et al., 2016). Given the high prevalence of sexual choking, which is typically enacted as a form of strangulation and thus has serious implications for risk of stroke, cardiac arrest, airway collapse, damage to the larynx, loss of consciousness, and brain injury (Herbenick et al., 2022d; Patch et al., 2018), clinicians might consider providing their patients with anticipatory guidance about this sexual practice. Given the higher prevalence of consensual and nonconsensual rough sex behaviors reported by young adults as well as by sexual minoritized individuals, continuing education efforts might prioritize those working in LGBTQ+ health, college health, and in family planning clinics.

Third, our findings have implications for sexuality educators, health educators, and those engaged in violence prevention. Educators might integrate findings from this study in their college human sexuality courses, community presentations, or workforce development courses for clinicians. Findings from our study might serve as a springboard for talking with learners about the importance of clear and explicit consent. Educators might also engage with learners in understanding the types of risk involved for various rough sex practices, especially given the online misinformation about some sexual practices, such as sexual choking/strangulation (Balle et al., 2024; Herbenick et al., 2023b).

Finally, given the high prevalence of sexual choking and its risks of injury and death, it is time for public health agencies to address sexual choking. In 2008, following media reports of unintentional strangulation deaths among youth who had played the “choking game” the Centers for Disease Control addressed this form of “choking”/strangulation (Centers for Disease Control & Prevention, 2008). As part of their guidance, they indicated that parents, educators, and healthcare providers needed to become aware of the choking game and its warning signs among youth; that there needed to be better mortality surveillance; and that research was needed in order to identify youth awareness of and engagement in the choking game as well as both risk and protective factors. Given that sexual choking has become far more prevalent than the choking game ever was, and that it poses risks of injury and death and yet has been rife with misinformation, public health agencies have an important role to play in helping to educate the public and prevent harm. Such education will need to be done in ways that are non-stigmatizing, medically accurate, and attentive to the complexities of gender, power, pleasure, and sexuality.

## Conclusion

In conclusion, we found that most U.S. adults ages 18 to 94 reported having ever engaged in at least one form of the rough sex practices assessed in this study, though there was an age cohort effect with younger adults being more likely than older adults to report having engaged in or experienced each of the sexual practices assessed. We found that reports of rough sex varied significantly by age, gender, and sexual orientation identity, with sexual minoritized adults being more likely to report rough sex practices as compared with heterosexual adults. Clinicians and educators need to be aware of these shifts in sexual behavior. Also, continued population-level surveillance of diverse sexual practices is needed as is public health leadership on the issue of sexual choking.

## Declarations

### Data Availability

Contact the authors to request.
